# Integrated Ultrasound‐Enrichment and Machine Learning in Colorimetric Lateral Flow Assay for Accurate and Sensitive Clinical Alzheimer's Biomarker Diagnosis

**DOI:** 10.1002/advs.202406196

**Published:** 2024-09-19

**Authors:** Shuqing Wang, Yan Zhu, Zhongzeng Zhou, Yong Luo, Yan Huang, Yibiao Liu, Tailin Xu

**Affiliations:** ^1^ School of Biomedical Engineering College of Chemistry and Environmental Engineering The Institute for Advanced Study (IAS) Shenzhen University Shenzhen Guangdong 518060 P. R. China; ^2^ Beijing Key Laboratory for Bioengineering and Sensing Technology University of Science and Technology Beijing Beijing 100083 P. R. China; ^3^ Longgang District Central Hospital of Shenzhen Shenzhen Guangdong 518116 P. R. China

**Keywords:** alzheimer's disease, colorimetric biosensing, lateral flow assay, machine learning, ultrasound enrichment

## Abstract

The colloidal gold nanoparticle (AuNP)‐based colorimetric lateral flow assay (LFA) is one of the most promising analytical tools for point‐of‐care disease diagnosis. However, the low sensitivity and insufficient accuracy still limit its clinical application. In this work, a machine learning (ML)‐optimized colorimetric LFA with ultrasound enrichment is developed to achieve the sensitive and accurate detection of tau proteins for early screening of Alzheimer's disease (AD). The LFA device is integrated with a portable ultrasonic actuator to rapidly enrich microparticles using ultrasound, which is essential for sample pre‐enrichment to improve the sensitivity, followed by ML algorithms to classify and predict the enhanced colorimetric signals. The results of the undiluted serum sample testing show that the protocol enables efficient classification and accurate quantification of the AD biomarker tau protein concentration with an average classification accuracy of 98.11% and an average prediction accuracy of 99.99%, achieving a limit of detection (LOD) as sensitive as 10.30 pg mL^−1^. Further point‐of‐care testing (POCT) of human plasma samples demonstrates the potential use of LFA in clinical trials. Such a reliable lateral flow immunosensor with high precision and superb sensing performance is expected to put LFA in perspective as an AD clinical diagnostic platform.

## Graphical abstract

The lateral flow assay platform is integrated with a portable ultrasound‐driven device for sample pretreatment to improve the limit of detection to 10.30 pg mL^−1^ in undiluted serum, followed by machine learning algorithms to achieve 98.11% accuracy in differentiation and 99.99% accuracy in quantification for point‐of‐care Alzheimer's diagnosis.

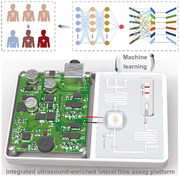

## 1 Introduction

AD, an ongoing threat to human well‐being, is the most common neurodegenerative disorder characterized by severe cognitive impairment.^[^ ^1^ ^]^ Given the lack of effective eradication means to date, it is crucial to enable timely intervention and treatment for early diagnosis.^[^ ^1^, ^2^ ^]^ The gold standard of AD diagnosis is the quantitative detection in cerebrospinal fluid (CSF).^[^ ^3^, ^4^, ^5^ ^]^ However, CSF samples are obtained by lumbar puncture, an invasive procedure prone to pain and health complications.^[^ ^6^ ^]^ Compared to CSF detection, blood biopsy is a less‐invasive, easy‐to‐use, and low‐cost emerging technology that has been applied to detect blood biomarkers for initial screening.^[^ ^7^, ^8^, ^9^ ^]^ There is strong evidence that tau proteins, when abnormally aggregated and hyperphosphorylated in patients, may serve as an alternative AD blood biomarker for predicting disease progression.^[^ ^10^, ^11^ ^]^ However, tau proteins are present in blood at extremely low levels and require a highly sensitive analytical platform for detection. Therefore, the development of a sensitive, accessible, and reliable tool for detecting tau proteins in blood is of great interest for the early detection of AD.

The LFA is widely recognized as an excellent POCT platform for blood analysis due to its affordability, simplicity, short turnaround time, and user‐friendliness.^[^ ^12^, ^13^, ^14^, ^15^, ^16^ ^]^ LFA has been instrumental in the rapid diagnosis of AD, and its combination with magnetic enrichment, Raman, and electrochemical techniques has enabled the highly sensitive detection of tau proteins and other biomarkers.^[^ ^17^, ^18^, ^19^ ^]^ However, these assays typically have limitations such as complex sample preparation, high cost, and the need for specialized analytical equipment, so there is still a long way to go to achieve practical applications. Among the many LFA methods available, the colloidal AuNP‐based lateral flow immunoassay has a unique advantage in that the colorimetric signals generated by the aggregation of AuNPs can be read quickly by the naked eye, making it highly cost‐effective for qualitative testing and offering great potential for a large‐scale community screening of AD.^[^ ^20^, ^21^, ^22^, ^23^ ^]^ However, the major developmental bottleneck of the conventional AuNP‐based LFA is its inability to detect complicated body fluids with high sensitivity and sufficient accuracy. This limitation restricts its application from laboratory to clinical diagnosis.^[^ ^24^, ^25^, ^26^ ^]^ Nowadays, ML algorithms are coming to the fore in rapid diagnosis and precision medicine.^[^ ^27^ ^]^ Such algorithms can automate the processing and analysis of diverse biological data, adjust the accuracy and efficiency of the model by continuously learning from the data, and have been shown to perform quite well in the analysis of LFA.^[^ ^28^, ^29^ ^]^ Despite some progress in intelligent detection, accurate quantitative analysis in LFA is poorly explored for ML‐based Alzheimer's diagnosis.

To address the above challenges of sensitive detection and accuracy, we developed an ML‐optimized LFA with ultrasound‐induced nanoparticle enrichment for ultra‐trace detection of AD biomarker tau proteins. By controllably adjusting the input frequency, the acoustic field within the ultrasound device effectively promoted the aggregation and reaction of the target proteins with the capture probes. The combination of KNN (K‐nearest neighbor) and GPR (Gaussian process regression) achieved effective differentiation with 98.11% accuracy and accurate prediction with 99.99% accuracy for the enhanced chromatographic signals of the undiluted serum samples. We also performed direct quantitative and qualitative classification experiments on human plasma samples, respectively, to investigate the reliability of this integrated LFA platform on real samples. In conclusion, our work provides a new ultra‐traceable, low‐cost, sensitive‐responsive, and well‐detected method for the detection of tau proteins, which may be conducive to the advancement of POCT screening for AD.

## 2 Results and Discussion

### 2.1 Machine Learning‐Optimized Integrated LFA System with Ultrasound Enrichment

**Figure 1:**
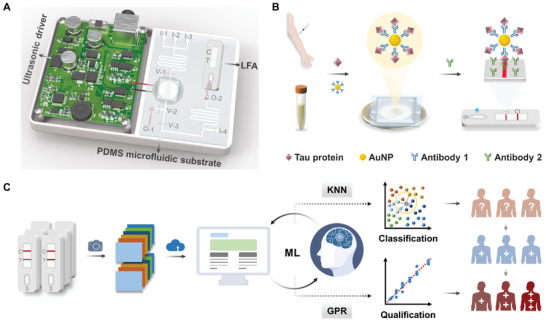
Overview of the developed machine learning (ML)‐optimized lateral flow assay with ultrasound enrichment for tau protein biosensing in Alzheimer's disease (AD). A) Schematic of the integrated ultrasound‐enriched lateral flow assay (LFA) device. I‐1/2/3/4, inlet 1/2/3/4; V‐1/2/3, valve 1/2/3; O‐1/2, outlet 1/2; S, sample port; T, test line; C, control line. B) Schematic of the detection strategy with corresponding immunostructure. AuNP, colloidal gold nanoparticle. C) Schematic of the ML‐optimized LFA colorimetric analysis of tau protein classification and qualification for AD monitoring. KNN, K‐nearest neighbor; GPR, Gaussian process regression.

**Figure** **1**A depicts the integrated ultrasound‐enriched LFA device for the sensitive detection of tau proteins for early monitoring of AD. The device consists of an ultrasound driver as the power source, a poly (dimethyl siloxane) (PDMS) microfluidic substrate as the enrichment platform (Figure S1, Supporting Information), and an LFA test strip as the analytical tool. First, AuNPs were modified with capture antibodies (Ab_1_) to form the capture probes. The collected serum sample and AuNP‐Ab_1_ probes were added separately to inlet 1 and inlet 2 of the microfluidic substrate, mixed through the microtubule channel, and then sonicated for enrichment in the cylindrical PDMS chamber above the piezoelectric transducers (PZT). When valve 1 and valve 2 were turned on, the AuNP‐Ab_1_‐Tau conjugate and uncaptured probes aggregated in the center of the cavity in the presence of ultrasound. Cleaning buffer (PBS) was then added to the inlet 3 to wash the substances three times and the washed aggregates were redissolved in the running buffer (PBS + 4% BSA) for further LFA (Figure S2, Supporting Information).

After the release of the ultrasonically pre‐treated sample on the test strip, the enriched conjugate in the sample is subsequently recognized by the detection antibody (Ab_2_) immobilized on the test line, resulting in a sandwich structure of AuNP‐Ab_1_‐Tau‐Ab_2_ complex with high specificity and sensitivity (Figure 1B). Due to the continuous accumulation of the AuNP‐Ab_1_‐Tau‐Ab_2_ complex, a red specific band visible to the naked eye appeared on the test lines of the strip. Finally, we photographed the target area with a camera, obtained the corresponding RGB values as a raw database, and uploaded them to the computer where they were analyzed by ML algorithms to enable effective classification and accurate quantification of tau proteins for early AD monitoring (Figure 1C).

### 2.2 Characterization of the Ultrasound‐Enriched LFA

**Figure 2:**
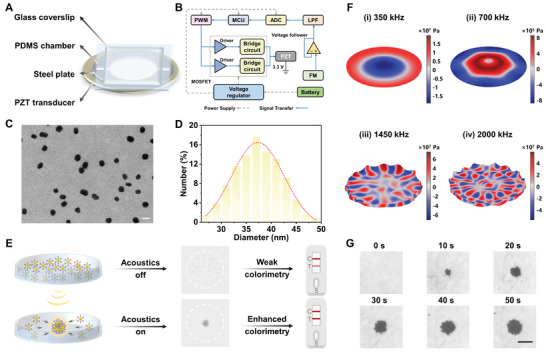
Characterization of the ultrasound‐enriched biosensing system. A) Structure of the integrated ultrasound‐driven platform. B) Schematic design of the driver circuit. FM, frequency modulation; LPF, low‐pass filter; ADC, analog‐digital converter; MCU, micro‐control unit; PWM, pulse width modulation; PZT, piezoelectric transducer; MOSFET, metal‐oxide‐semiconductor field‐effect transistor. C) Transmission electron microscopy image (scale bar: 50 nm) and D) size distribution of AuNPs (mean diameter 37.3 ± 0.2 nm, n = 130). E) Principle of the ultrasound‐enriched LFA. F) Numerical simulation of the sound pressure distribution in the PDMS cavity at different input frequencies, including 350, 700, 1450, and 2000 kHz. G) Time‐lapse micrographs of AuNPs’ aggregation (≈1 × 10^−8^ mol L^−1^) in the microcavity. Scale bar: 100 µm.

Ultrasound‐enhanced particle aggregation technology is particularly suited to the detection of ultra‐trace biomarkers for clinical diagnosis due to its non‐contact nature, excellent manipulability, and rapid response.^[^ ^30^, ^31^ ^]^ In contrast to traditional methods such as membrane filtration^[^ ^32^ ^]^ and magnetic separation,^[^ ^33^, ^34^, ^35^ ^]^ ultrasound pretreatment offers a new direction of non‐contact external actuation and has been successfully applied in some related work in our group.^[^ ^36^, ^37^, ^38^, ^39^, ^40^, ^41^, ^42^ ^]^ The integrated ultrasound‐driven platform comprises a glass coverslip, a PDMS chamber, a steel plate, and a PZT (**Figure** **2**A). To enhance the suitability of the device for POCT, a schematic of the driver circuit was designed and a portable printed circuit board with dimensions of ≈7.5^*^6^*^2.5 cm was fabricated using standard design procedures based on the schematic (Figure 2B; Figure S3, Supporting Information).

AuNPs were used as colorimetric tracers for LFA because of their visibility to the naked eye, the simplicity of the synthesis method, and the low cost of production. Typically, AuNPs used in LFA are smaller than 50 nm in size, as larger particles are less stable and consume more labeled antibodies.^[^ ^43^, ^44^ ^]^ To obtain the best colorimetric signal for the ultrasound‐enrichment mode, we used AuNPs of 20, 30, and 40 nm for comparison, respectively (Figures 2C,D; Figure S4, Supporting Information). Figure S5 (Supporting Information) displayed an elevation in the zeta potential and a redshift of the maximum absorption peak of AuNP‐Ab_1_, suggesting the successful modification of the capture antibody on the AuNPs’ surface. The mixed solution of AuNP‐Ab_1_ and tau proteins was introduced into the microcavity of the integrated ultrasound‐driven platform. Receiving a specific frequency signal from the driver circuit, the PZT generated continuous acoustic waves via the inverse piezoelectric effect. The acoustic wave then passed through the steel plate and acted on the PDMS cavity where a differential pressure field was produced to achieve the controlled actuation and enrichment of the nanoparticles. In contrast to the general Brownian motion of particles without ultrasound treatment, the enriched particles exhibit a clear aggregation state in the center of the microcavity. The swarming of microparticles promoted rapid binding of specific particles and simultaneous enrichment of the target, which enabled the LFA test strips to completely absorb the concentrated microlite samples within the chromatographic time, thereby enhancing the colorimetric signal of LFA (Figure 2E). Under the same experimental conditions, 40 nm AuNPs produced a stronger colorimetric intensity, ≈4 and 6 times that of 20 and 30 nm, respectively (Figure S6, Supporting Information). Therefore, 40 nm AuNPs were chosen as the final tracers in combination with Ab_1_ to specifically capture the target proteins in this work.

The high‐frequency voltage output from the drive circuit is converted by PZT into ultrasonic signals which are reflected and superimposed in the reaction chamber to form a non‐linear standing wave acoustic field. The acoustic radiation force is an acoustic swimming phenomenon that induces the dynamic motion of particles by exerting a non‐zero summed force through the non‐linear standing wave acoustic field. In essence, the acoustic radiation force can be divided into the primary radiation force (PRF) and the secondary radiation force (SRF).^[^ ^45^, ^46^ ^]^ While the SRF is typically negligible, the PRF is the major driving force behind the controlled aggregation of particles, which can be expressed as Equation 1:^[^ ^47^ ^]^

(1) $$F_{PRF}=-\left(\frac{\pi P_{a}^{2}V_{p}\beta_{m}}{2\lambda }\right)\cdot \Phi \left(\beta ,\;\rho \right)\cdot \sin\left(2kx\right)$$

(2) $$\Phi \left(\beta ,\;\rho \right)=1+\frac{3\left(\rho_{p}-\rho_{m}\right)}{2\rho_{p}\;+\;\rho_{m}}-\frac{\beta_{p}}{\beta_{m}}$$

where *P*_a_ and *x* are the acoustic pressure amplitude and the distance to the pressure node on the wave propagation axis, *λ*, and *k* are the wavelength and the wavenumber, *ρ*_p,_ and *ρ*_m_ are the density of the particles and the medium, *β*_p,_ and *β*_m_ are the compressibility of the particles and the medium, respectively, and *V*_p_ is the volume of the particles. Thus, the acoustic radiation force is not only a function of the particle size and the amplitude of the acoustic field but also of the acoustic contrast factor (*Φ*) of the particle. AuNPs and their couplings can be considered as rigid particles (*β*_p_/*β*_m_→0) and are of higher density and lower compressibility compared to the aqueous medium (*Φ*(*β*, *ρ*)>0). This indicates that the direction of the PRF is aligned with the node, leading to an aggregation of particles along the node direction. The acoustic pressure distribution inside the microcavity was simulated under different input frequencies by the finite element analysis model (see the Supporting Information for detailed analysis). As shown in Figure 2F, the acoustic pressure field formed only a single pressure node at a specific frequency matched to the resonant cavity, while at other frequencies it was irregularly distributed with multiple nodes, thereby corroborating the theoretical feasibility of our portable ultrasonic device for particle aggregation at the resonant frequency. To clarify the practical verification of the assembly process, we introduced AuNPs into the reaction chamber in an aqueous solution. When a frequency of 700 kHz was input, the experimental results showed that AuNPs rapidly formed a settled and dense assembly within 50 s (Figure 2G and Video S1, Supporting Information), indicating that acoustic radiation forces generated by uneven ultrasonic pressure distribution could drive nanoparticles to migrate toward acoustic pressure nodes.

### 2.3 Machine Learning‐Optimized LFA

It is challenging for traditional data processing methods to extract valid biomarker information and analyze complex data networks for precise diagnosis.^[^ ^48^ ^]^ With the increasing popularity of artificial intelligence, ML algorithms are proving to be faster, stronger, more flexible, and more accurate in processing redundant data and analyzing key parameters.^[^ ^49^ ^]^ To ensure the precision of the diagnostic model on a restricted dataset, an advanced ML approach was applied to investigate the LFA detection system with integrated ultrasound enrichment for high‐accuracy diagnosis.

**Figure 3:**
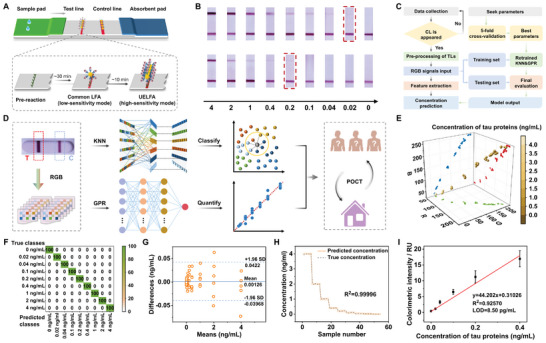
Machine learning‐based LFA for AD point‐of‐care testing (POCT). A) Design and signal enhancement mechanism of the LFA test strip. The arrow indicates the flow direction of the sample. UELFA, ultrasound‐enriched LFA. B) Optical images of LFA test strips corresponding to tau protein gradient concentrations (0–4 ng mL^−1^) before and after ultrasound enrichment. The images in the top row are after ultrasound enrichment and the images in the bottom row are before ultrasound enrichment. C) Procedures of colorimetric signal processing on the dataset. D) Flowchart of tau protein biosensing by KNN classification and GPR quantification. E) 3D plot of the RGB signals from test lines with a gradient of tau protein concentrations of the PBS samples. The mapped colors red, green, and blue represent the R, G, and B values, respectively. F) KNN confusion matrix for the discrimination of tau proteins in the PBS samples based on the concentration classes. G) Bland‐Altman plot between predicted and true concentration values of the PBS samples based on GPR analysis. H) Signal response between predicted and true concentration values of the PBS samples based on GPR analysis. I) Linear correlation between analyte concentration and colorimetric intensity within the range of 0–0.4 ng mL^−1^ (n = 3) tau proteins in the PBS samples.

Following ultrasonic enrichment, the sample was added to the sample pad port of the LFA strip and then flowed through the nitrocellulose (NC) membrane by capillary action. The AuNP‐Ab_1_ probes were specifically recognized by the pre‐cured capture antibodies on the NC membrane to form the test lines, while the uncaptured AuNP‐Ab_1_ continued to flow forward and reacted with the secondary antibody to form the control lines, indicating the validity of the assay results. Compared to the absence of pre‐treatment, the target exhibited a rapid enrichment following sonication, which significantly increased the probability of being captured by Ab_2_ per unit of time. This resulted in ultrasound‐enriched LFA being a high‐sensitivity model (**Figure** **3**A). Using the presence or absence of ultrasound enrichment as a variable, we prepared a series of LFA test strips with graded concentrations (0–4 ng mL^−1^) of tau proteins in PBS. The color depth of the test lines exhibited a gradual decline with decreasing tau protein concentrations, with 0.2 ng mL^−1^ representing the approximate threshold for visual detection before ultrasound enrichment and 0.02 ng mL^−1^ representing the approximate threshold for visual detection after ultrasound enrichment, suggesting that the acoustic enrichment treatment achieved an ≈10‐fold colorimetric enhancement (Figure 3B).

The data obtained from the bands by ultrasound‐enriched LFA were collected and filtered according to the presence or absence of the control lines. RGB signals were extracted from the test lines at each concentration gradient to form a raw database. In the model training phase, 5‐fold cross‐validation was employed to evaluate the ML model. This process involves randomly dividing the original data into five equal subsets, then sequentially selecting one of the subsets as the testing set and the other four as the training sets. Subsequently, the model was trained and evaluated five times on the testing set to enhance its generalizability. Finally, the retrained KNN and GRP models were optimized and applied to the concentration prediction of the database (Figure 3C). On the one hand, the KNN classification algorithm calculated the distance (Euclidean distance of the RGB value) between the samples to be classified and each sample in the training set, determined the class to which the samples belonged based on the closeness of the distance and classified the sample according to the concentration classes.^[^ ^50^ ^]^ On the other hand, the GPR regression algorithm fitted a normal distribution to the known concentrations through a Gaussian process, yielded a flexible function capable of predicting the unknown concentrations, and achieved quantification of low abundance detectors (Figure 3D).^[^ ^51^ ^]^

The possibility of the ML‐optimized pattern was first explored by analyzing the results of samples in the PBS matrix. To achieve the optimal signal‐to‐noise ratio for the colorimetric signals, the relevant parameters in LFA were optimized (Figures S7–S11, Supporting Information). Based on the optimal parameters, a series of LFA test strips with graded tau protein concentrations was prepared, the test lines of which were captured and converted to the corresponding RGB database (Figure S12, Supporting Information). The RGB values of the test lines demonstrated a tendency to increase with decreasing concentration (Figure 3E), in accordance with the principles of double‐antibody‐sandwich immunology.^[^ ^52^ ^]^ In our work, colorimetric data features were extracted and effectively categorized by the KNN classifier. The confusion matrix is a 2D array used to evaluate the performance of the KNN classification algorithm. The accuracy in classifying the dataset is expressed as a percentage in the matrix, with correct classifications on the main diagonal and misclassifications elsewhere. As shown in Figure 3F, the RGB values corresponding to different tau protein concentrations in the PBS samples can be discriminated in the KNN confusion matrix with an average accuracy of 100%. Compared to the traditional analysis method fitted by Euclidean distance and target concentration, the proposed KNN classification model significantly improved the correlation of the data by 25.97% (Figure S13, Supporting Information). Meanwhile, the RGB signals were fed into the GPR model, which outputs a concentration prediction. The consistency of the predicted values with the true values was verified by the Bland‐Altman plot. As depicted in Figure 3G, there were more than 94% of the data points fell within the limits of agreement, indicating a high degree of agreement between the predicted and actual values. The correlation coefficient (R^2^) between the two reaches 0.99996, demonstrating that the GPR model has excellent quantitative power (Figure 3H). Compared with the traditional analysis method fitted by R, G, and B values and target concentration, the proposed GPR quantification model greatly improved the correlation of the data by 18.40, 27.22, and 5.52%, respectively (Figure S14, Supporting Information). Based on the calibration curve and the IUPAC recommendation,^[^ ^53^, ^54^ ^]^ the LOD for tau proteins in PBS was calculated by extrapolating the concentration from the optical background signals plus its three times standard deviation (0.0022 ± 0.0021 ng mL^−1^) and was found to be as low as 8.50 pg mL^−1^ (Figure 3I; Figure S15 and Table S1, Supporting Information). This value suggests that the detection of low abundant tau proteins can be achieved by a colorimetric method, demonstrating that our program is a breakthrough with potential applications. From the above, the ML‐supported synergistic analysis greatly facilitated efficient classification and accurate quantification of tau proteins for AD POCT.

### 2.4 Sensitive and Accurate Diagnosis of AD

**Figure 4:**
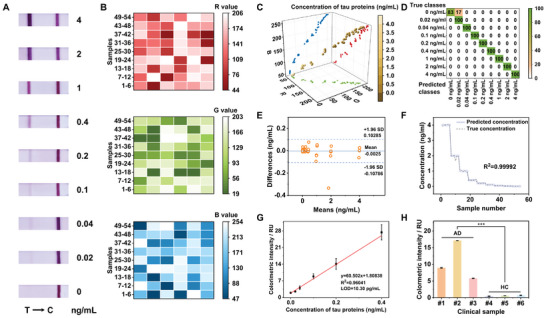
Ultrasound‐enriched and machine learning‐assisted LFA for clinical Alzheimer's sample testing. A) Optical images of LFA test strips corresponding to tau protein gradient concentrations (0–4 ng mL^−1^) of the serum samples. B) Heat map showing the R, G, and B results of tau protein detection of the serum samples. C) 3D plot of the RGB signals from test lines with a gradient of tau protein concentrations of the serum samples. The mapped colors red, green, and blue represent the R, G, and B values, respectively. D) KNN confusion matrix for the discrimination of tau proteins in the serum samples based on the concentration classes. E) Bland‐Altman plot between predicted and true concentration values of the serum samples based on GPR analysis. F) Signal response between predicted and true concentration values of the serum samples based on GPR analysis. G) Linear correlation between analyte concentration and colorimetric intensity within the range of 0–0.4 ng mL^−1^ (n = 3) tau proteins in the serum samples. H) Statistical significance analysis of the colorimetric intensity in AD and HC clinical plasma samples (two‐tailed Student's t‐test; ^***^, *p* < 0.001; AD, Alzheimer's disease patients; HC, healthy controls).

To explore the clinical applicability of the developed scheme, we have investigated the potential of our biosensing system in the context of real‐world samples. To simulate serum samples from AD patients, artificial undiluted serum samples were prepared by spiking tau‐free serum samples with varying concentrations of tau proteins (0.02, 0.04, 0.1, 0.2, 0.4, 1, 2, and 4 ng mL^−1^). As shown in **Figure** **4**A, the color depth of the test lines gradually decreased with decreasing tau protein concentration, with 0.02 ng mL^−1^ representing the approximate threshold for visual detection, consistent with the phenomenon of the PBS samples. The test lines of the serum samples were collected and converted to the corresponding RGB database, demonstrating a tendency to increase with decreasing concentration (Figure 4B,C).

Similar to the model training on the PBS sample database, colorimetric data features extracted from the serum samples were effectively categorized by the KNN classifier. Except for a bias in signal discrimination between 0.02 and 0 ng mL^−1^, the majority of RGB values corresponding to different tau protein concentrations can be discriminated with an average accuracy of 98.11% in the KNN confusion matrix (Figure 4D). Compared to the traditional analysis method fitted by Euclidean distance and target concentration, the proposed KNN classification model significantly improved the correlation of the data by 30.03% (Figure S16, Supporting Information). The GPR quantifier then took over the task of predicting concentrations. As depicted in Figure 4E, more than 98% of the data points were within the limits of agreement, indicating a high degree of agreement between the predicted and actual values. The R^2^ between the two reaches 0.99992, showing that the GPR model still has a strong predictive ability in serum sample testing (Figure 4F). Compared with the traditional analysis method fitted by R, G, and B values and target concentration, the proposed GPR quantification model greatly improved the correlation of the data by 20.66, 23.60, and 33.44%, respectively (Figure S17, Supporting Information).

The linear fit curve of tau protein was obtained based on the GPR model, with a linear dynamic range (LDR) of 0–0.4 ng mL^−1^ and a slope of 0.96041, reflecting a strong positive correlation (Figure 4G; Figure S18 and Table S2, Supporting Information). The limit of detection (LOD) for tau proteins in undiluted serum was calculated to be as low as 10.30 pg mL^−1^ by extrapolating the concentration from the optical background signals plus its three times standard deviation (0.0025 ± 0.0026 ng mL^−1^). This value is slightly lower than that of the PBS matrix due to the fact that the serum matrix is more complex and contains other interfering proteins. However, it is still capable of detecting tau proteins at very low concentrations, suggesting that our protocol still has considerable potential. Compared to previously reported AD sensing methods (Table S3, Supporting Information), our scheme exhibits several advantages, including low sample consumption, rapid response, a short detection time, and ease of use. To further evaluate the performance of our strategy, a specificity experiment was conducted to avoid cross‐reactivity. The significant difference in colorimetric intensity (*p *< 0.001) showed that non‐target samples did not generate specific signals that interfered with the required analysis (Figure S19, Supporting Information).

Finally, six clinical plasma samples were measured to assess the clinical performance. The corresponding RGB signals (Figure S20, Supporting Information) were fed into the trained model for fitting and were output as predicted concentration values. Based on the reported authoritative work^[^ ^55^ ^]^ which concluded clinically relevant concentrations of tau proteins in blood plasma, the output concentrations were divided into concentration intervals corresponding to patients and healthy individuals to make qualitative conclusions about the presence or absence of AD. To validate the accuracy of the AD diagnostic model, we compared the results with the hospital diagnosis and colorimetric analysis using a commercially available colloidal gold immunoassay analyzer. Concentration outputs and interval comparisons were performed separately on the six blinded samples, three of which were ultimately identified as positive, while the other three were negative, consistent with the disease diagnosis given by the hospital (Table S4, Supporting Information). Subsequently, we ran the measured LFA bands through the commercial colloidal gold immunoassay analyzer and analyzed the colorimetric intensity for significance. Statistical significance analysis of the AD and HC clinical plasma samples showed a significant difference (*p* < 0.001) in colorimetric intensity, indicating that our method has a high degree of accuracy in differentiating the complex clinical samples (Figure 4H).

## 3 Conclusion

In summary, we present a machine learning‐optimized lateral flow assay with ultrasound‐driven nanoparticle enrichment for the detection of AD biomarker tau proteins. The acoustic fields within the microcavity contributed significantly to the controlled accumulation of tau proteins with capture probes through the programmable input frequency. Subsequently, we employed the KNN algorithm as a classifier and the GPR algorithm as a quantifier, achieving 98.11% accuracy in differentiation and 99.99% accuracy in quantification against undiluted samples. The proof‐of‐concept study demonstrated the potential application of the method in clinical biospecimens, with a salient distinction confirmed in clinical plasma samples. However, further efforts are required to be invested in the user‐friendliness of the platform for population‐based mass screening. One avenue for future exploration is the development of a mobile application that would enable users to access their personal health information on time. The integration of machine learning algorithms and ultrasound technology could facilitate the advancement of lateral flow immunology, paving the way for the emergence of intelligent and integrated diagnostics.

## 4 Experimental Section

### Materials

Colloidal gold nanoparticles (AuNPs, 40 nm), nitrocellulose (NC) membrane, sample pad, bottom plate (PVC) and absorbent pad were purchased from Jiening (Shanghai, China). Cover glass was purchased from Citotest Scientific Co., Ltd (Nanjing, China). Bovine serum albumin (BSA), sucrose, and phosphate buffer saline (0.01 m PBS, pH 7.4) were provided by Sigma–Aldrich (St. Louis, MO). Goat anti‐mouse IgG (bs‐0296G) was purchased from Bioss (Beijing, China). Tau monoclonal antibody (Ab_1_, 5B10) was purchased from Novus Biologicals (Colorado, USA). Tau monoclonal antibody (Ab_2_, HT7) was obtained from Thermo Fisher Scientific (Massachusetts, USA). Recombinant human tau_441_ protein (ab84700), Aβ_40,_ and Aβ_42_ were purchased from Abcam (Cambridge, UK). All relevant reagents were used without further purification and were prepared by dilution with ultrapure water unless otherwise stated.

### Apparatus

The XYZ platform and the programmable strip cutter used to cut 4 mm wide strips were supplied by Jiening. The piezoelectric transducers (PZT) were provided by Harbin Core Tomorrow Science and Technology Co., Ltd. China. The real‐time movement of the particles was recorded by an Eclipse Ni microscope device equipped with a DS‐Ri2 microscope camera and a 4X objective (Nikon, Japan). The morphologies of AuNPs were obtained with transmission electron microscopy (TEM, HT7700, Hitachi, Japan). The UV–vis absorption spectrum was recorded using a GENESYS 10S spectrophotometer (Thermo Fisher Scientific, USA) and the zeta potential was measured using a Zetasizer Nano ZS analyzer (Malvern, UK). Photographs of the LFA test strips were taken by a Nikon Z5 camera (Tokyo, Japan). The colloidal gold immunoassay analyzer was provided by Henan Guanyu Co., Ltd. China.

### Construction of the Ultrasound‐Driven Integrated Device

The ultrasound‐driven integrated device was designed as follows: A poly (dimethyl siloxane) (PDMS) microcavity (diameter = 5 mm, thickness = 0.25 mm) fabricated by soft lithography adhered to the surface of the PZT ceramic plate with a conductive gel. The PZT ceramic chip was then integrated into the 3.3 V ultrasound driver board through the attachment of copper wires. The ultrasonic driver board, including battery, voltage follower, and metal oxide semi‐conductor field effect transistor (MOSFET), was used as a signal generator and amplifier to obtain high‐frequency voltages. The PZT ceramic plate acted as a transducer to convert the electrical signals into ultrasonic signals, which were then transmitted through the PDMS film and super‐imposed into the microcavity to generate standing‐field acoustic waves, allowing programmable manipulation of the nanoparticles in the microcavity.

### Preparation of the LFA Test Strips

The lateral flow device consisted of four components: sample pad, NC membrane, absorbent pad, and adhesive backing pad. Glass fibers were selected as sample pads, fully immersed in a buffer solution (pH 7.5) containing 2% BSA, 2% sucrose, and 0.25% Tween‐20, and then dried at 37 °C for 5 h. Goat anti‐mouse IgG prediluted in PBS and Ab_2_ were sprayed onto the NC membrane as control and test lines (7 mm apart) respectively, and then the NC membrane was dried at 37 °C for 1 h. Finally, the pads were well assembled on the base plate with a 2 mm overlap each and cut to 4 mm width for storage at 4 °C for further use.

### Detection of Tau Proteins by Machine Learning‐Optimized LFA with Ultrasound Enrichment

First, a mixture of Ab_1_‐modified AuNPs and target samples with varying concentrations of tau proteins was injected into the microcavity of the integrated platform. The detection process was initiated by switching on the instrument, which accelerated the formation of immune sandwich structures via ultrasonic oscillations within the sound pressure field. After 10 min, the output frequency was adjusted to 700 kHz to create a stable acoustic potential well for microparticle enrichment for 1 min. Finally, the target solution was injected out of the cavity and released to the LFA to obtain the results in 10 min. The LFA images were captured by a Nikon Z5 camera and converted into R, G, and B values as a raw database for algorithmic analysis.

### POCT of Tau Proteins in Clinical Samples

Serum samples and plasma samples were collected from the Longgang District Central Hospital of Shenzhen. This study was approved by the Longgang District Central Hospital of Shenzhen (Shenzhen, China) (ethics approval number: 2022ECPJ163). To evaluate the biosensing performance of the platform in real samples, tau proteins were added to human serum to mimic biological samples. Different concentrations of tau proteins were prepared with undiluted serum using the same measurement procedure as described above. Each experimental set was repeated three times. In addition, clinical plasma samples were diluted with PBS buffer (1/1 ratio) before testing and measured using a colloidal gold immunoassay analyzer to aid in the comparative analysis of the chromatographic results.

### Statistical analysis

All data obtained in this study were expressed as mean ± standard deviation and analyzed using Origin 2021 and Microsoft Office Excel. The sample size (n) for each statistical analysis was given in the figure legends or in the relevant part of the article. A finite element model was used to simulate the sound pressure distribution in the ultrasound field within the device by using Multiphysics software. Statistical significance analysis of the associated data was performed using the two‐tailed Student's t‐test. The probability (*P* valve) < 0.05 was considered statistically significant.

## Conflict of Interest

The authors declare no conflict of interest.

## Supporting information



Supporting Information

Supplemental Video 1

## Data Availability

The data that support the findings of this study are available in the supplementary material of this article.
