# Supplementary material for: Integrated Ultrasound‐Enrichment and Machine Learning in Colorimetric Lateral Flow Assay for Accurate and Sensitive Clinical Alzheimer's Biomarker Diagnosis
Source: Adv Sci (Weinh). 2024 Sep 19;11(42):2406196. doi: 10.1002/advs.202406196 (PMC11558096; doi:10.1002/advs.202406196)
Supplement: Supplementary file 1 — Supporting Information [file ADVS-11-2406196-s002.docx]

Supporting Information

Integrated Ultrasound-enrichment and Machine Learning in Colorimetric Lateral Flow Assay for Accurate and Sensitive Clinical Alzheimer's Biomarker Diagnosis

Shuqing Wang, Yan Zhu, Zhongzeng Zhou, Yong Luo, Yan Huang, Yibiao Liu, Tailin Xu*

S. Wang, Y. Zhu, Z. Zhou, T. Xu

School of Biomedical Engineering

College of Chemistry and Environmental Engineering

The Institute for Advanced Study (IAS)

Shenzhen University

Shenzhen, Guangdong 518060, P. R. China

E-mail: xutailin@szu.edu.cn

Y. Luo, Y. Huang

Beijing Key Laboratory for Bioengineering and Sensing Technology

University of Science and Technology Beijing

Beijing 100083, P. R. China

Y. Liu

Longgang District Central Hospital of Shenzhen

Shenzhen, Guangdong 518116, P. R. China

**Experiments**

**Preparation of AuNP-Ab_1_ capture probes**

The AuNP-Ab_1_ capture probes were synthesized according to the reported methods^[1]^ with the following modifications. First, 8 μL of 0.1 M K_2_CO_3_ solution and 10 μL of 1 mg mL^−1^ Ab_1_ were added sequentially to 1 mL of 40 nm AuNPs. The mixture was stirred at room temperature for 1 h. After the 1-hour incubation, 50 μL of 1% BSA in PBS was added and the mixture was blocked for 30 minutes at room temperature. After blocking, the solution was centrifuged in a tube at 7500 rpm for 30 minutes at 4℃, and the supernatant was then carefully aspirated. The scarlet pellet was resuspended in 100 μL of storage buffer (0.1 M PBS buffer, pH 8.0 with 5% BSA, 0.25% Tween-20, and 5% sucrose) and stored at 4°C in the dark until use.

**Parameter optimization of lateral flow immunochromatography**

To obtain better specific signals and to reduce the interference of the background signals, the signal-to-noise ratio method is adopted to optimize the colorimetric signals of the test lines. The parameters for optimization are the usage of capture antibody, the components of the running buffer, the usage of conjugates, and the usage of detection antibody (the spraying times of the test lines). The following is a brief description of the optimization steps using the example of optimizing the usage of capture antibody.

We set the spraying times of the test lines to 5×, the BSA content in the running buffer to 4%, the usage of conjugates to 5 μL, and designed the usage of capture antibody to have a gradient relationship (5, 15, 20, and 40 μg). 50 μL of 4 ng mL^−1^ tau proteins were added to the experimental groups, while a blank group without the proteins was used as a control. Three parallel groups were established, each controlling for the same experimental conditions. The specific signals of each experimental and blank group were measured by a colloidal gold immunoassay analyzer and divided to obtain three signal-to-noise ratios.

**Preparation of the poly (dimethyl siloxane) (PDMS) microfluidic substrate platform**

The PDMS microfluidic substrate platform (7.5×5×0.4 cm) was fabricated according to the reported methods^[2]^. A versatile plug microvalve for microfluidic application was used to achieve fluid control^[2]^. Based on the reported work using polylactic acid (PLA) rods with good biocompatibility and mechanical properties, we cut short rods of 4 cm length from PLA filaments and manually drilled a hole in the radial axis of the rod, followed by using a hole punch to make a through-hole at the corresponding position (intersecting with the microchannels) on the prepared PDMS microfluidic chip. Finally, the rod was inserted into the hole to align the hole with the microchannel. The pathway was opened when the extended end of the rod was parallel to the flow channel, while the fluid could not pass when it was perpendicular to the channel, thus achieving simple control of the fluid.

**Finite element model analysis**

Finite element analysis is an analytical method that uses the principle of mathematical approximation to simulate real physical systems by solving simpler problems (finite elements) instead of complex ones, thus approximating an infinite number of unknowns with a finite number of unknowns.^[3]^ In this work, this model was used to simulate the acoustic field pressure distribution in a microcavity based on the physical fields including the piezoelectric excitation sound field, electrostatics, and solid mechanics. Under the assumption that the pressure in the liquid domain varied harmonically with time, the ultrasonic pressure distribution is characterized by the Helmholtz equation (1) as follows:^[4]^

$$\begin{aligned} \text{∇}\text{ }\text{∙}\text{ }\text{(}\text{ ̶}\text{ }\frac{\text{1}}{\text{ρ}_{\text{0}}}\text{(}\text{∇}\text{p}\text{)) }\text{ ̶}\text{ }\frac{\text{v}^{\text{2}}\text{p}}{\text{ρ}_{\text{0}}\text{c}_{\text{s}}^{\text{2}}}\text{ =}\text{ 0 }\text{ }\text{ }\text{ }\text{ (}\text{1)} \end{aligned}$$

Where ∇, *ρ*_0_, *p*, v, c_s_ are the Laplace operator, the density of the medium, the acoustic variations of the platform pressure, the driving frequency of the ultrasound, and the speed of sound in the aqueous solution, respectively. The bottom surface of the piezoelectric transducer impedes the movement of the ultrasonic pressure in the z-direction, and this restriction is specified as a roller boundary condition which is added to the model. We have analyzed the interface between the piezoelectric transducer and the PDMS film. The normal component of this interface is used to realize the storage of samples in the microcavity and is represented by equation 2:

$$\begin{aligned} \text{n}\text{ }\text{∙ }\text{(}\frac{\text{1}}{\text{ρ}_{\text{0}}}\text{(}\text{∇}\text{p}\text{))}\text{ =}\text{ a}\text{n}\text{ }\text{ }\text{ }\text{ }\text{ }\text{ }\text{ }\text{ }\text{ }\text{ }\text{ }\text{ }\text{ (}\text{2}\text{)}\text{ } \end{aligned}$$

Where n is the normal vector of the outer surface of each unit and a_n_ is the normal acceleration component. Based on the above analysis of the model, the excitation frequency can be calculated to match a specific microcavity at the fixed parameters (*ρ*_0_, c_s_), and then the excitation frequency can be adjusted to achieve controllable adjustment of the sound pressure.


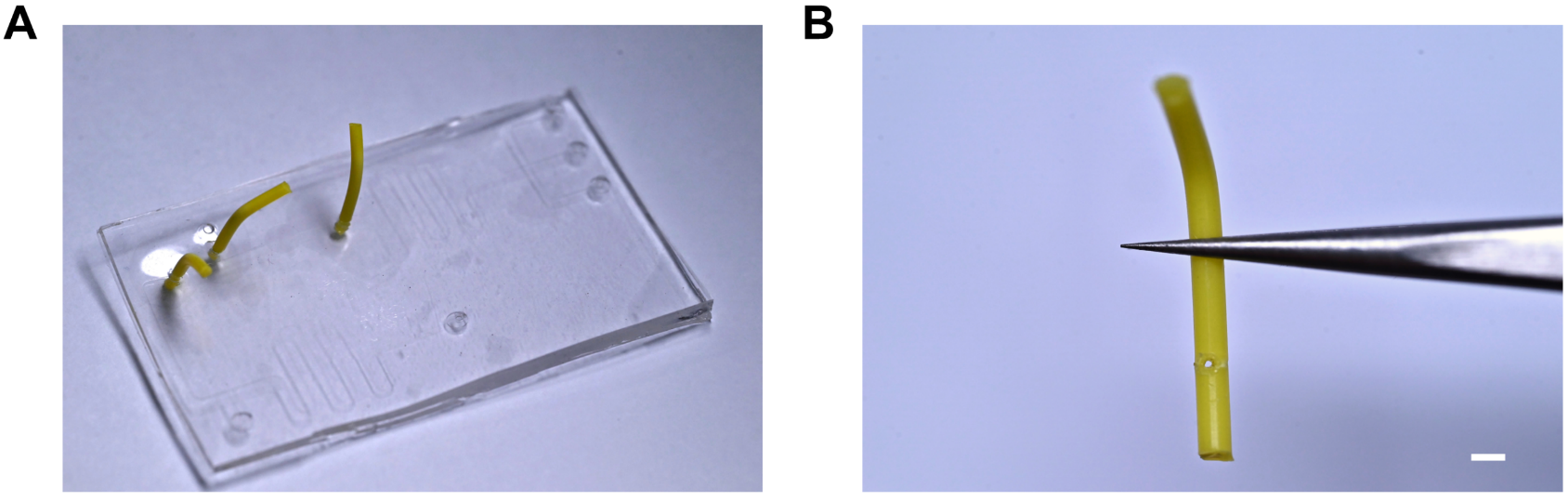


Figure S1. A) Physical drawing of the PDMS microfluidic substrate platform. B) Physical drawing of the versatile plug microvalve (the internal hole with 600 μm) for microfluidic applications. Scale bar: 1.75 mm.


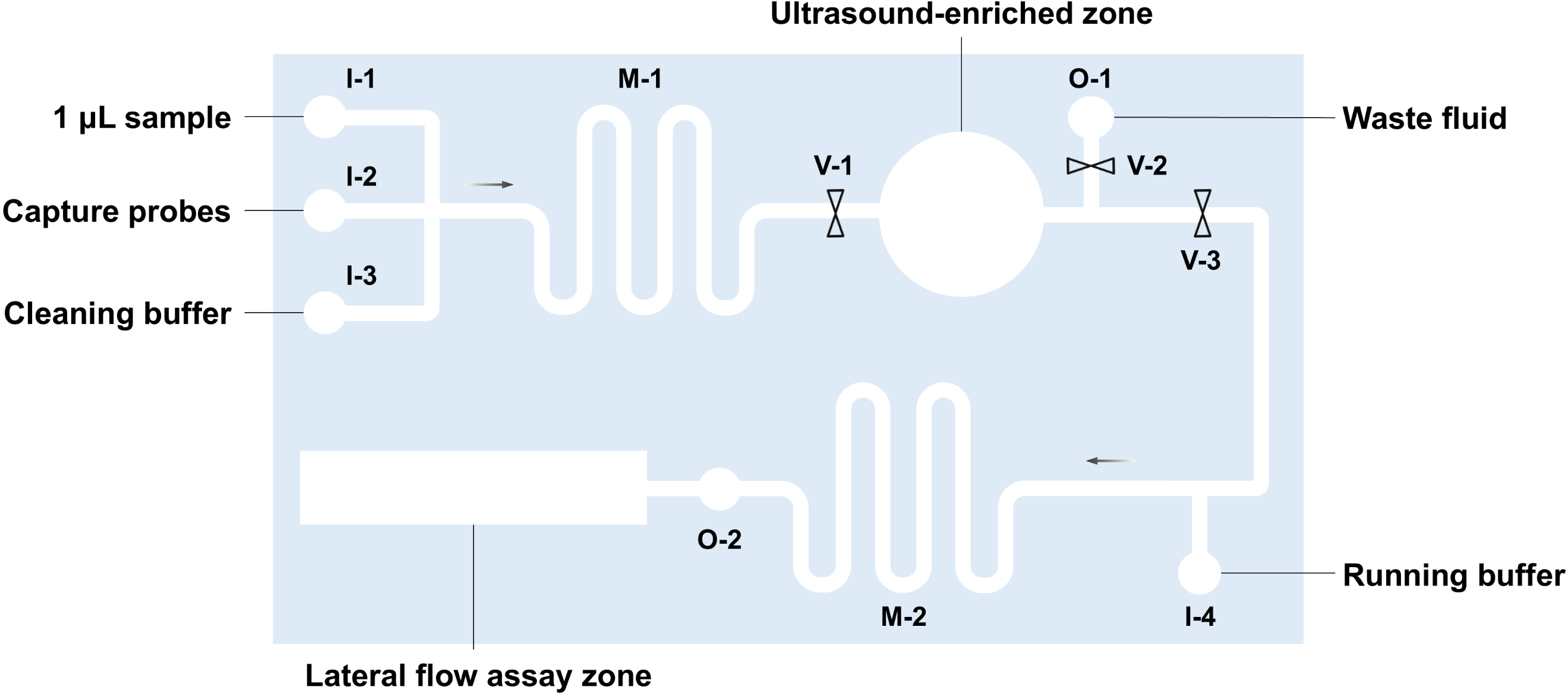


Figure S2. Design of the ultrasound-enriched LFA microfluidic system for tau protein detection. I-1/2/3/4, inlet 1/2/3/4; M-1/2, mixing zone 1/2; V-1/2/3, valve 1/2/3; O-1/2, outlet 1/2.


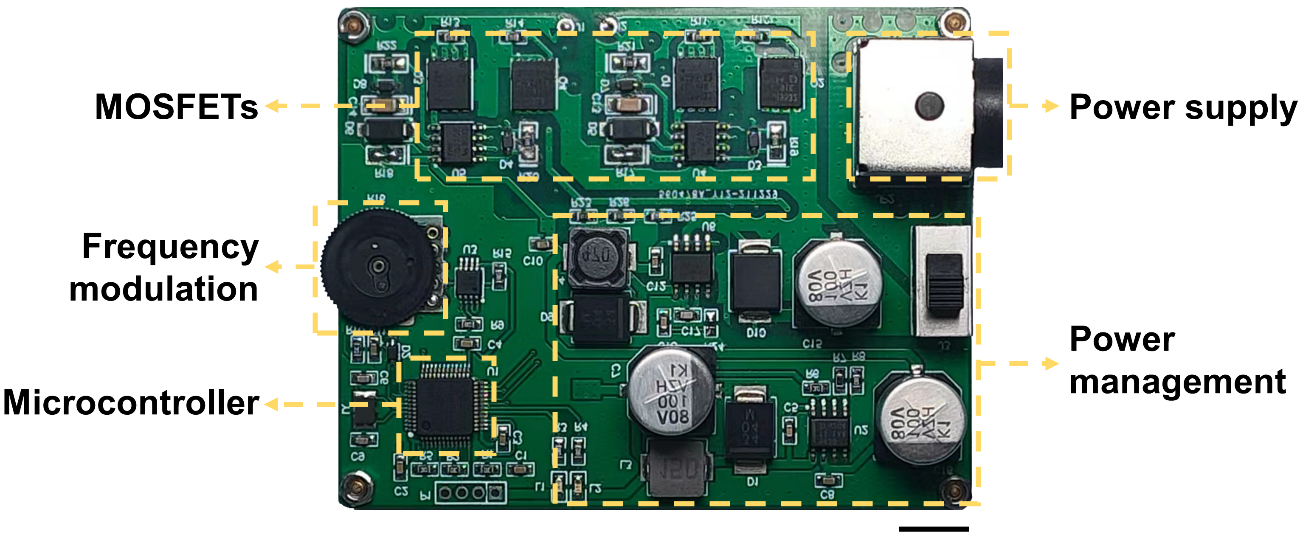


Figure S3. Physical drawing of the portable printed circuit board. Scale bar: 1 cm.


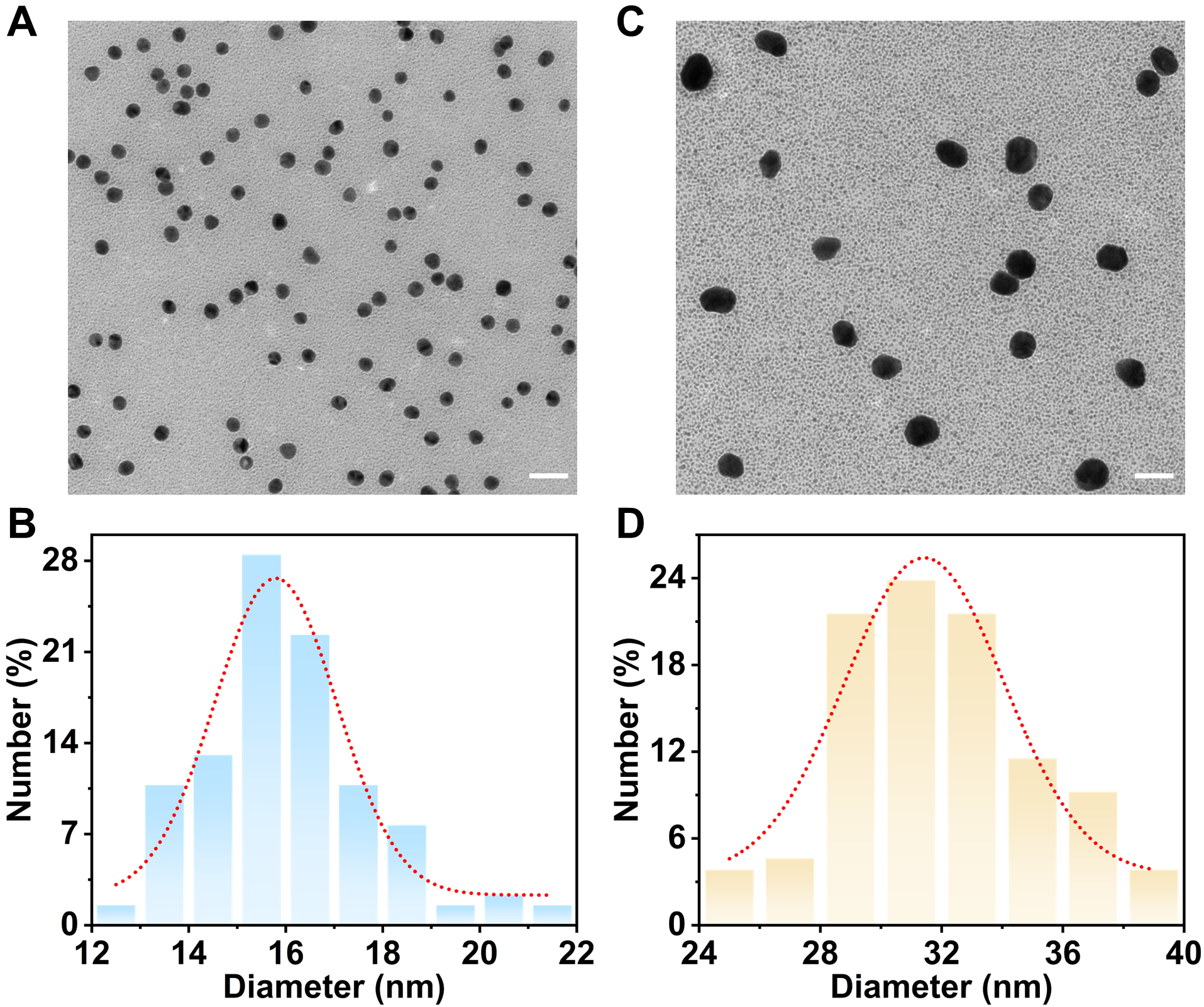


Figure S4. Characterization of AuNPs. A) Transmission electron microscopy image (scale bar: 50 nm) and B) size distribution of 20 nm AuNPs (mean diameter 15.8 ± 0.1 nm, n = 130). C) Transmission electron microscopy image (scale bar: 50 nm) and D) size distribution of 30 nm AuNPs (mean diameter 31.4 ± 0.4 nm, n = 130).


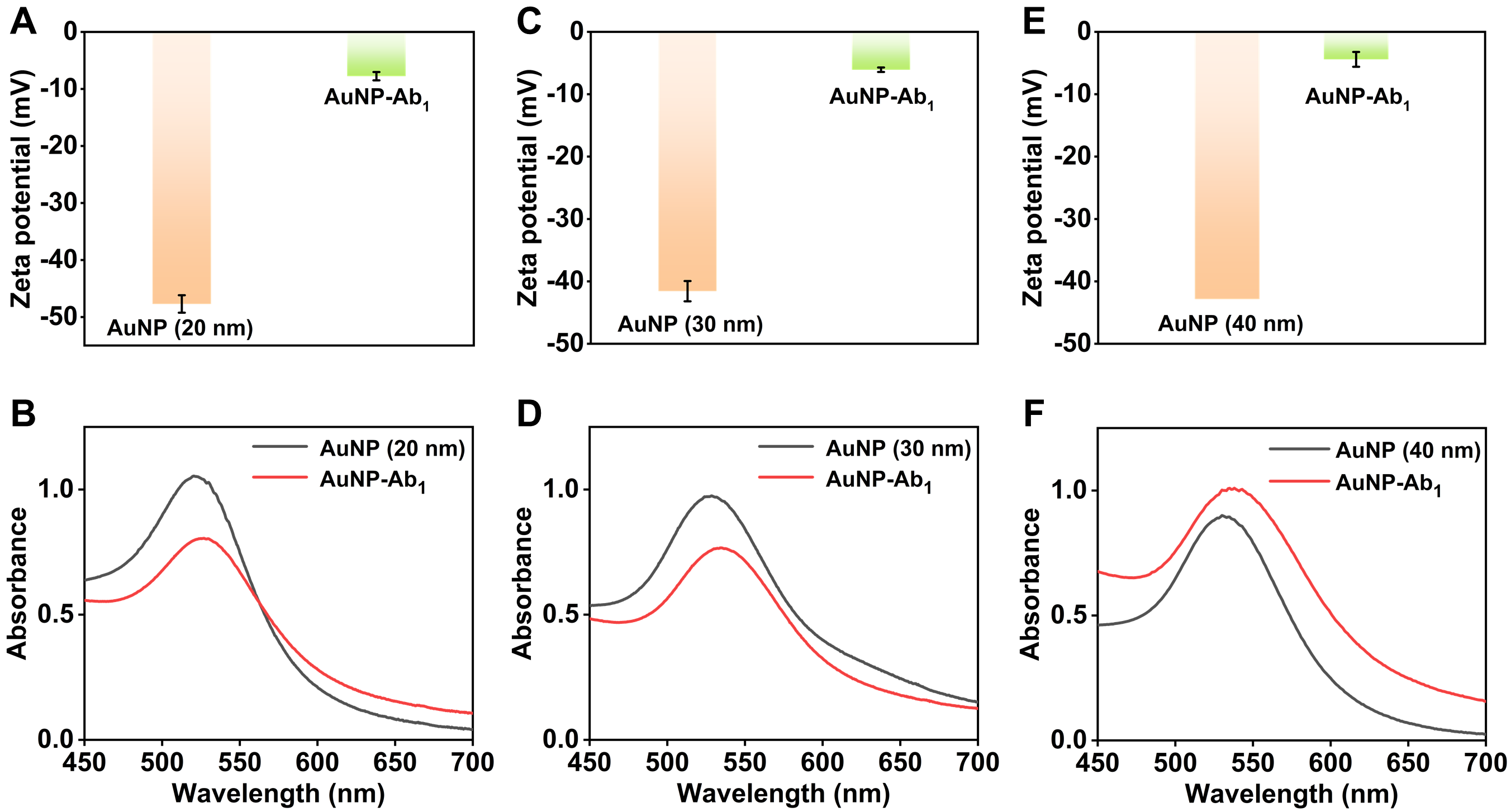


Figure S5. Characterization of nanoparticle conjugation. A) Zeta potential and B) UV-vis absorption spectra of AuNP (20 nm) and AuNP-Ab_1_. C) Zeta potential and D) UV-vis absorption spectra of AuNP (30 nm) and AuNP-Ab_1_. E) Zeta potential and F) UV-vis absorption spectra of AuNP (40 nm) and AuNP-Ab_1_.


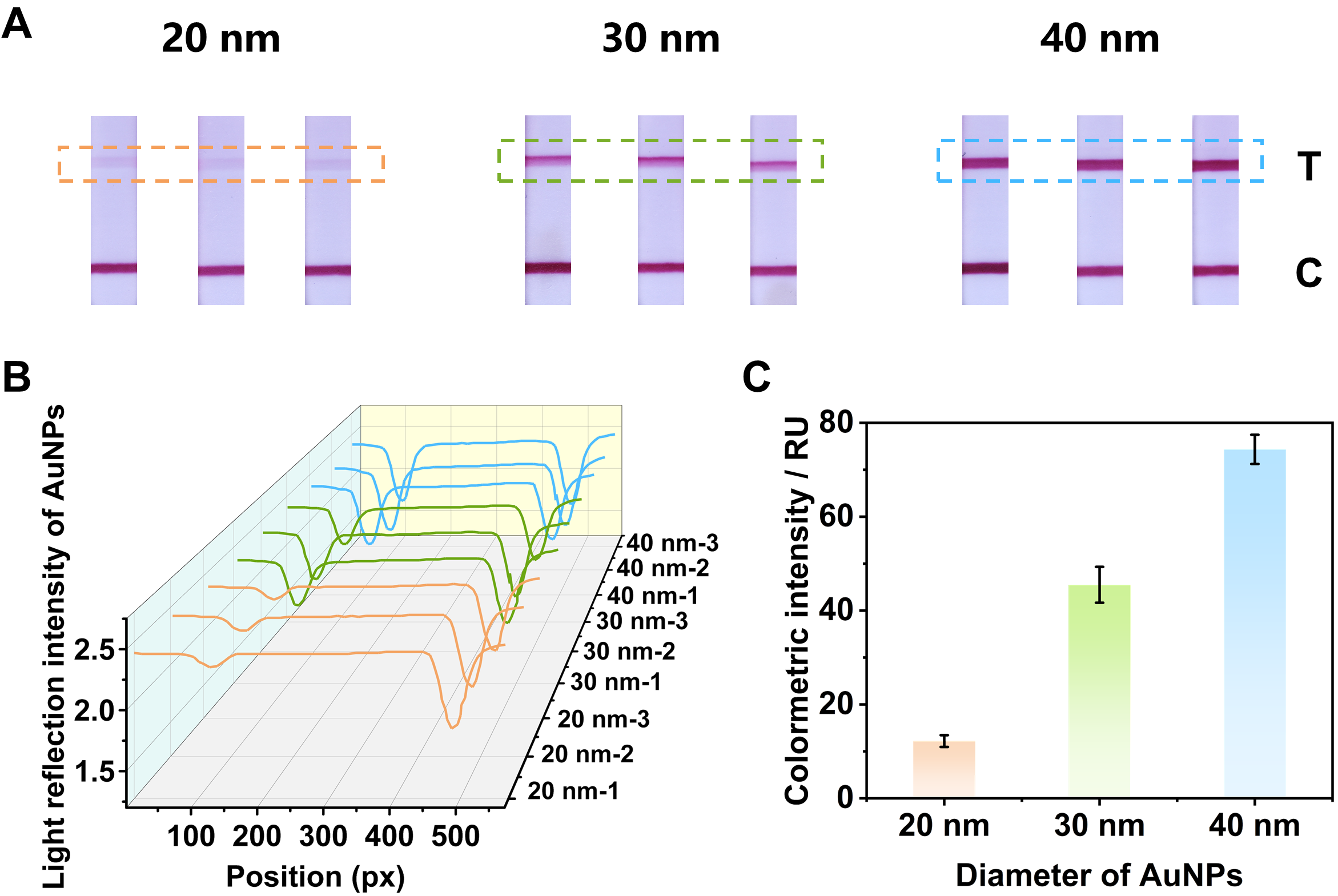


Figure S6. Characterization of AuNP diameter. A) Optical images of LFA test strips based on 20, 30, 40 nm AuNPs. B) Light reflection absorption mapping of 20 nm, 30, 40 nm AuNP-LFA test strips. C) Colorimetric intensity of 20 nm, 30, 40 nm AuNP-LFA test strips.


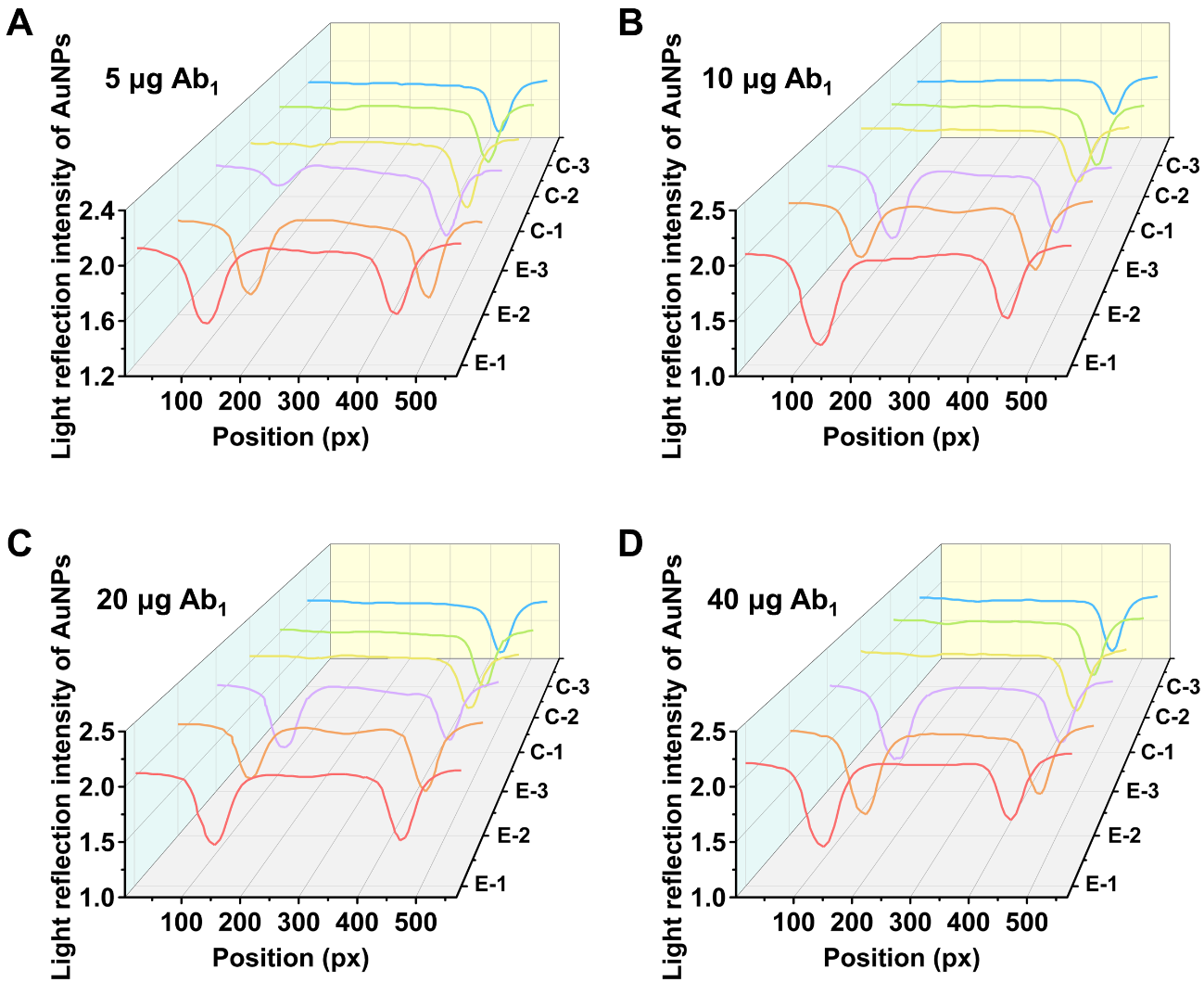


Figure S7. Parameter optimization of the usage of detection antibody (Ab_1_: 5, 10, 20, 40 μg) for the machine learning-optimized LFA platform with ultrasound enrichment for tau protein detection. E, experimental groups; C, control blank groups; Running buffer: 4% PBSB; Conjugate usage: 5 μL; Dispensing times of capture antibody: 5; Tau protein concentration: 4 ng mL^−1^.


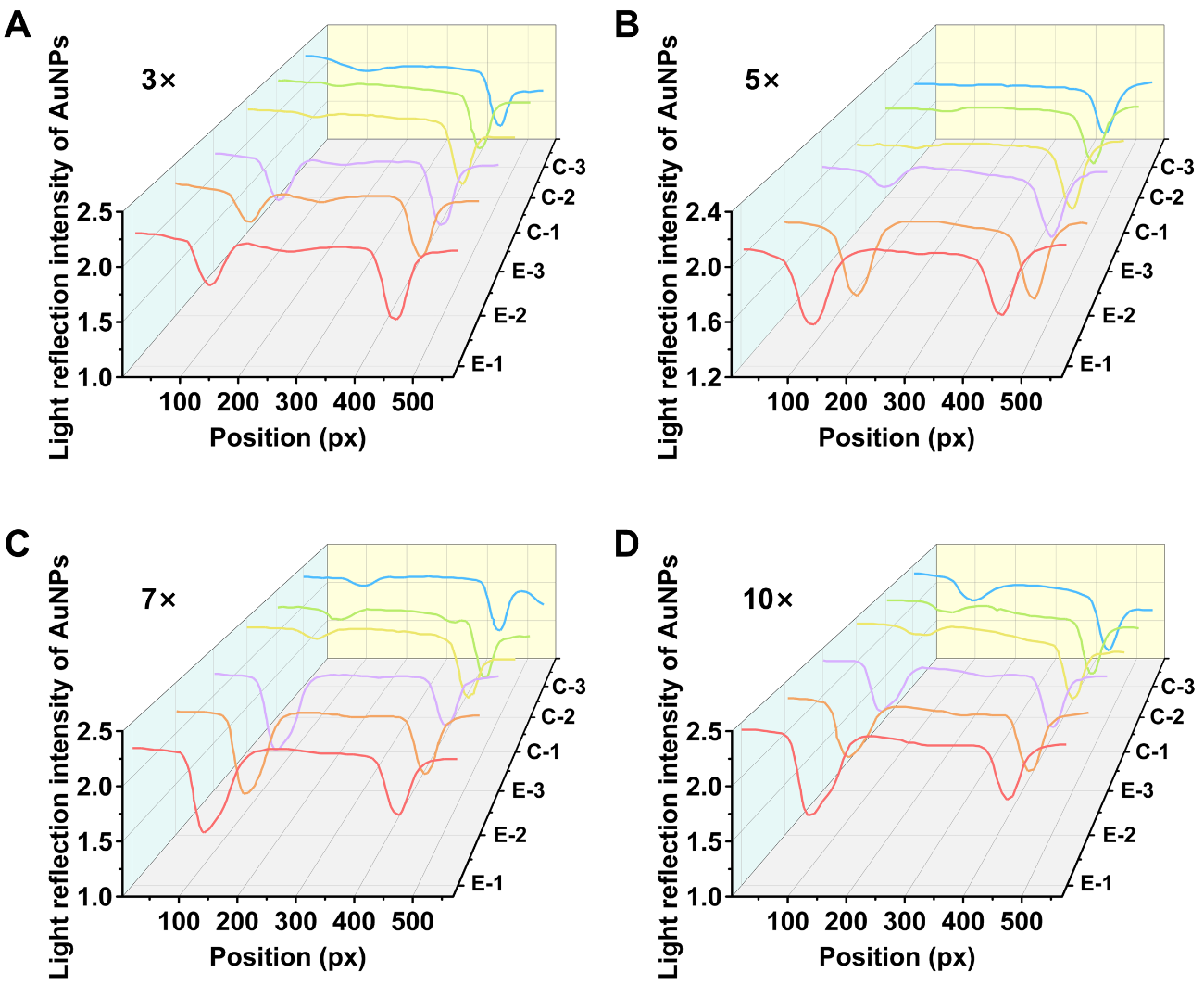


Figure S8. Parameter optimization of the usage of capture antibody (dispensing times: 3, 5, 7, 10) for the machine learning-optimized LFA platform with ultrasound enrichment for tau protein detection. E, experimental groups; C, control blank groups; Running buffer: 4% PBSB; Conjugate usage: 5 μL; Detection antibody in the conjugates: 5 μg; Tau protein concentration: 4 ng mL^−1^.


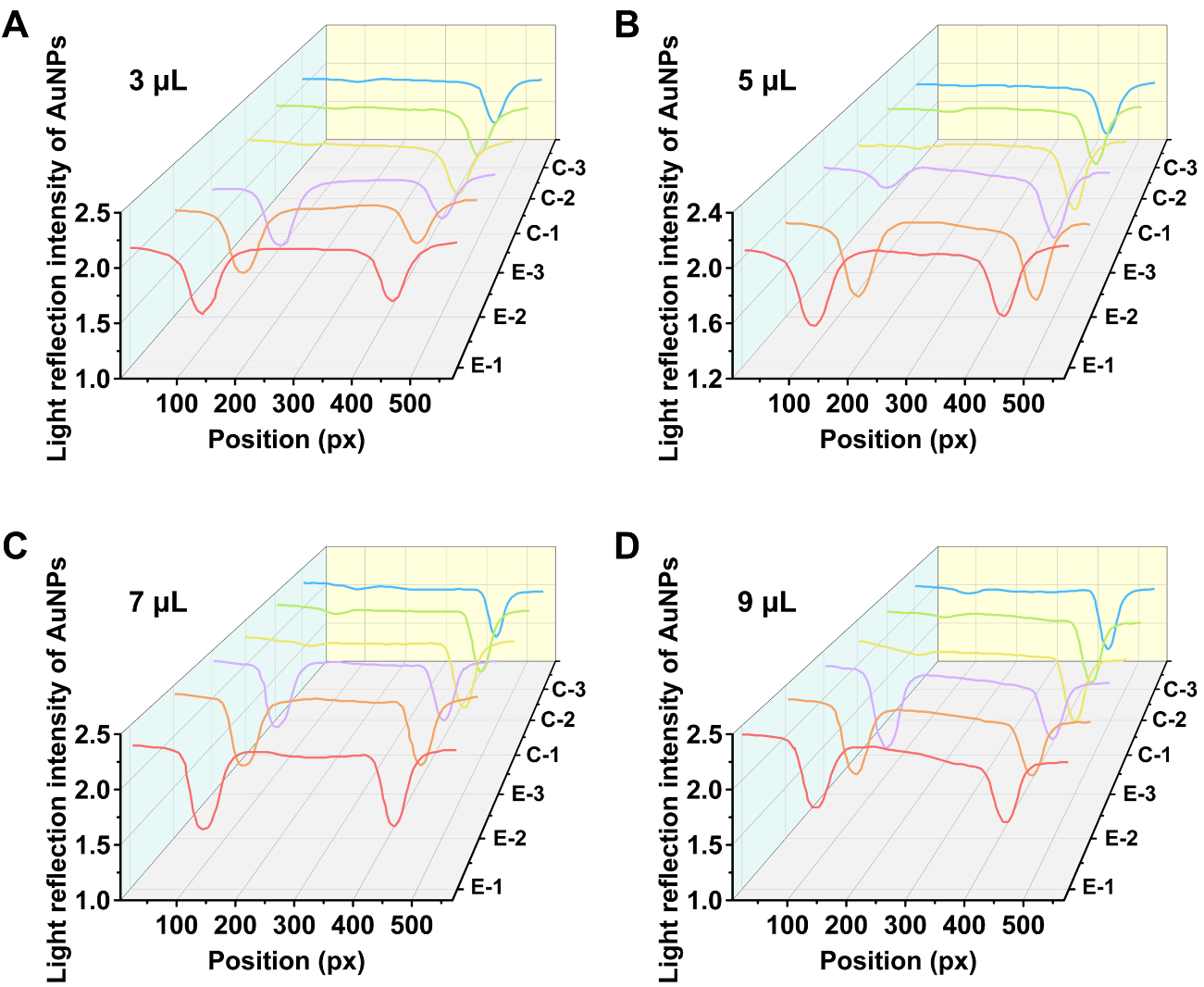


Figure S9. Parameter optimization of the conjugate usage (volume: 3, 5, 7, 9 μL) for the machine learning-optimized LFA platform with ultrasound enrichment for tau protein detection. E, experimental groups; C, control blank groups; Running buffer: 4% PBSB; Detection antibody in the conjugates: 5 μg; Dispensing times of capture antibody: 5; Tau protein concentration: 4 ng mL^−1^.


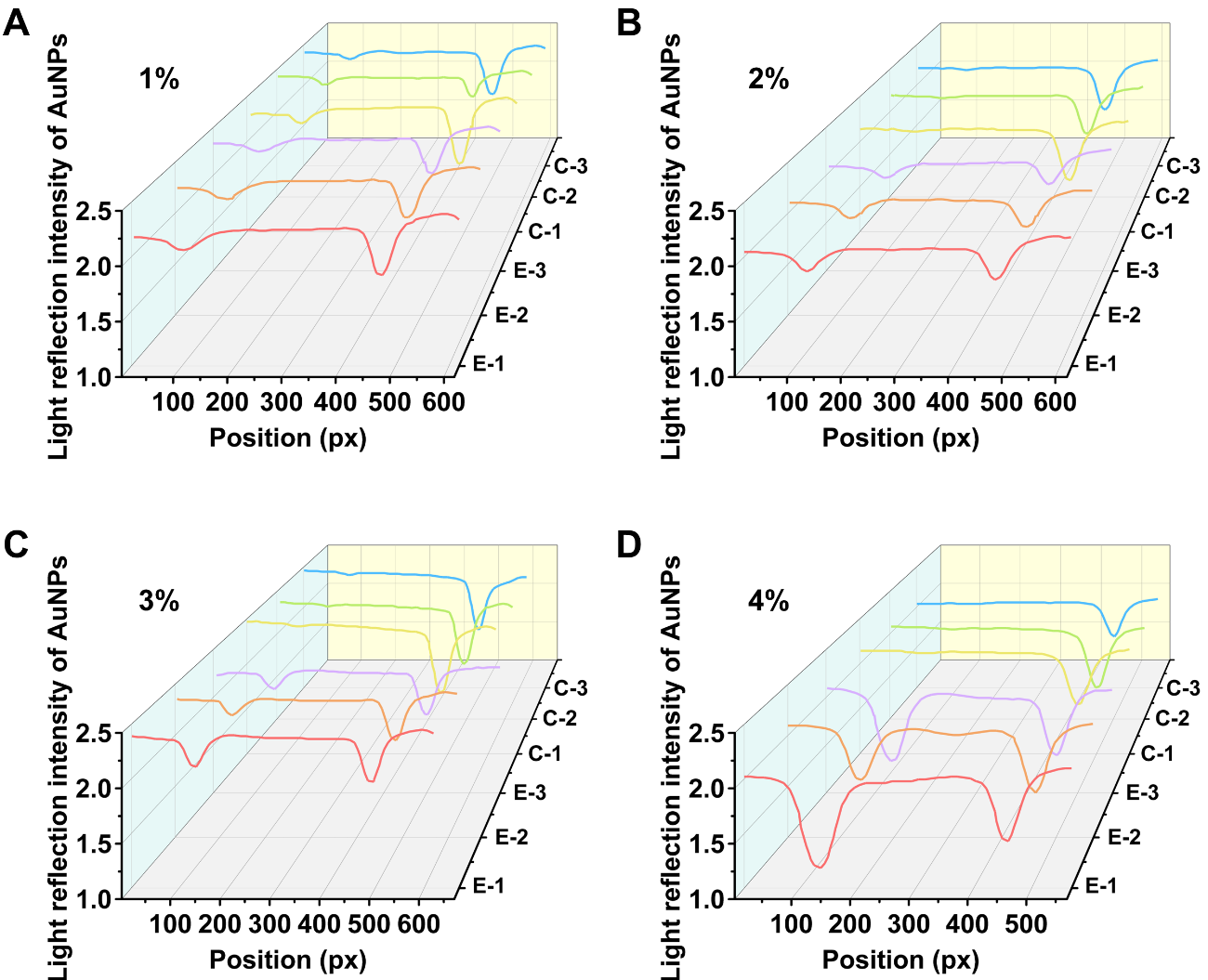


Figure S10. Parameter optimization of the running buffer (BSA usage: 1, 2, 3, 4%) for the machine learning-optimized LFA platform with ultrasound enrichment for tau protein detection. E, experimental groups; C, control blank groups; Conjugate usage: 5 μL; Detection antibody in the conjugates: 10 μg; Dispensing times of capture antibody: 5; Tau protein concentration: 4 ng mL^−1^.


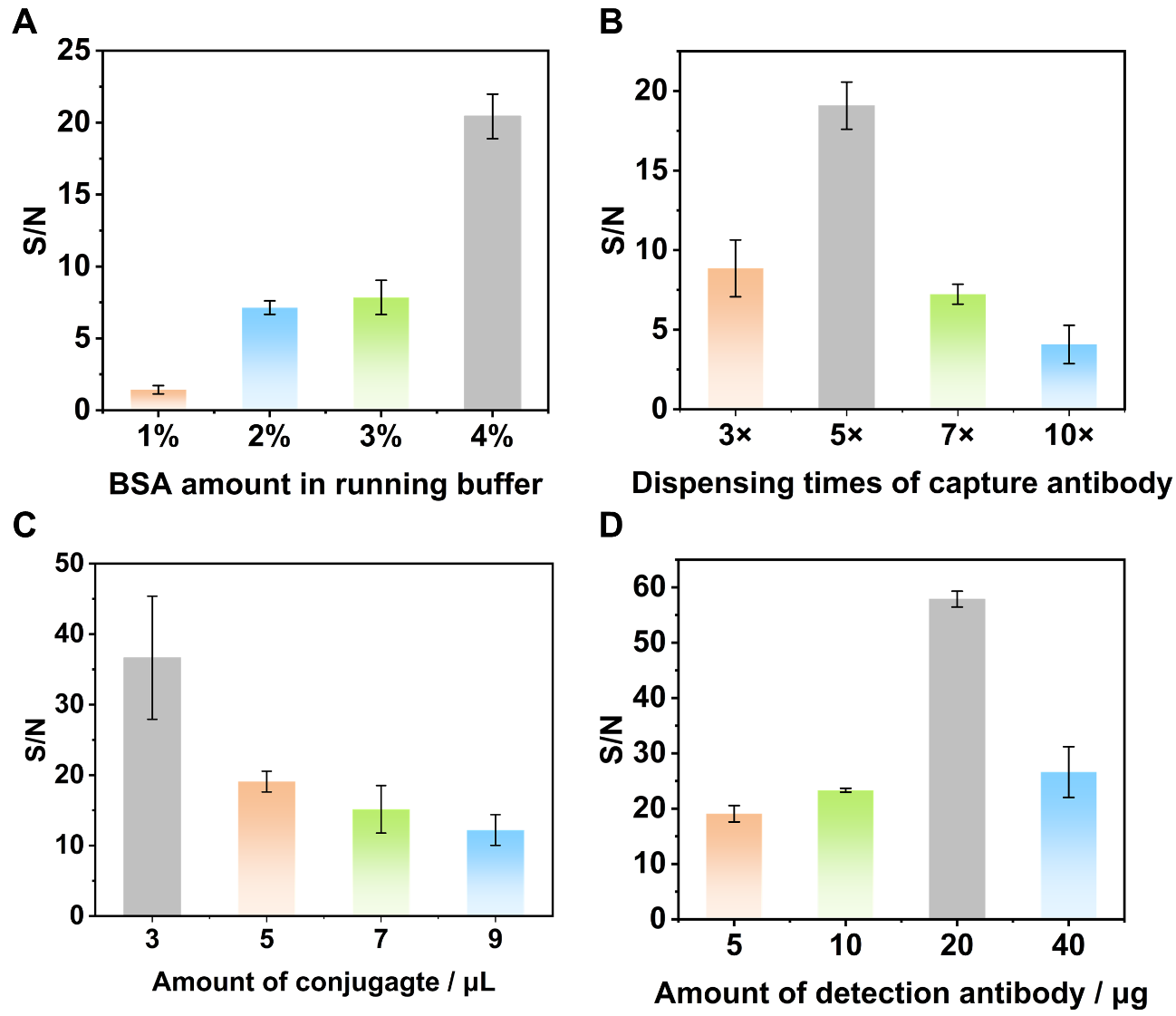


Figure S11. Parameter optimization on the signal-to-noise (S/N) ratio for the machine learning-optimized LFA platform with ultrasound enrichment for tau protein detection (n = 3). A) Effect of the running buffer. B) Effect of the dispensing times of capture antibody. C) Effect of the usage of conjugates. D) Effect of the usage of detection antibody.


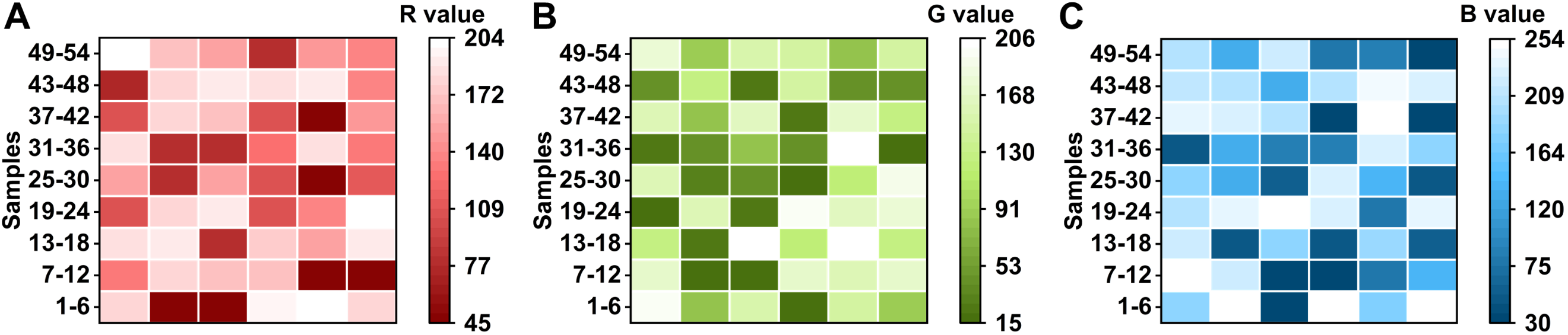


Figure S12. Heat map showing the R, G, B results of tau protein detection of the PBS samples.


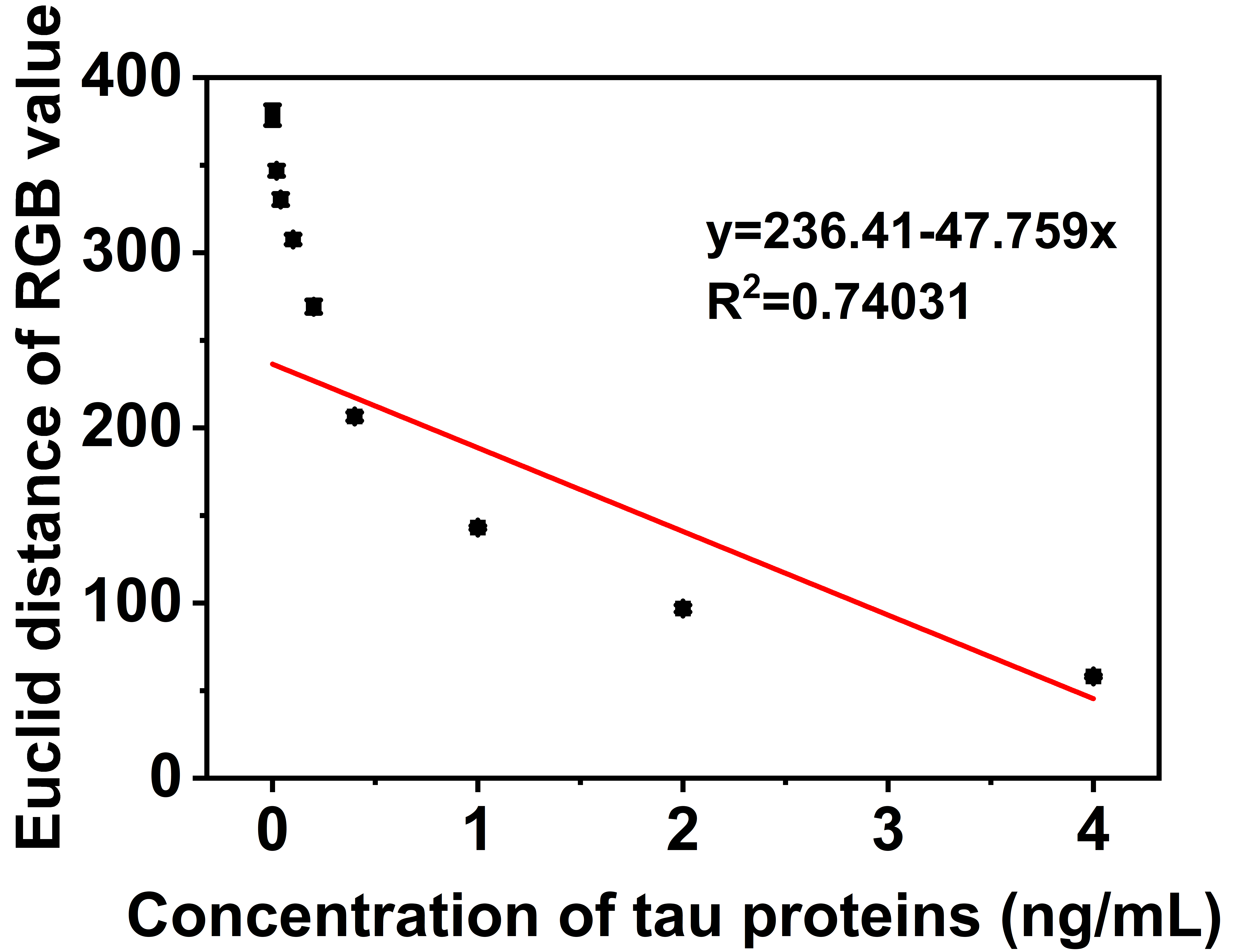


Figure S13. Linear fit curve of the RGB-valued Euclidean distance to the tau protein concentration in the PBS samples.


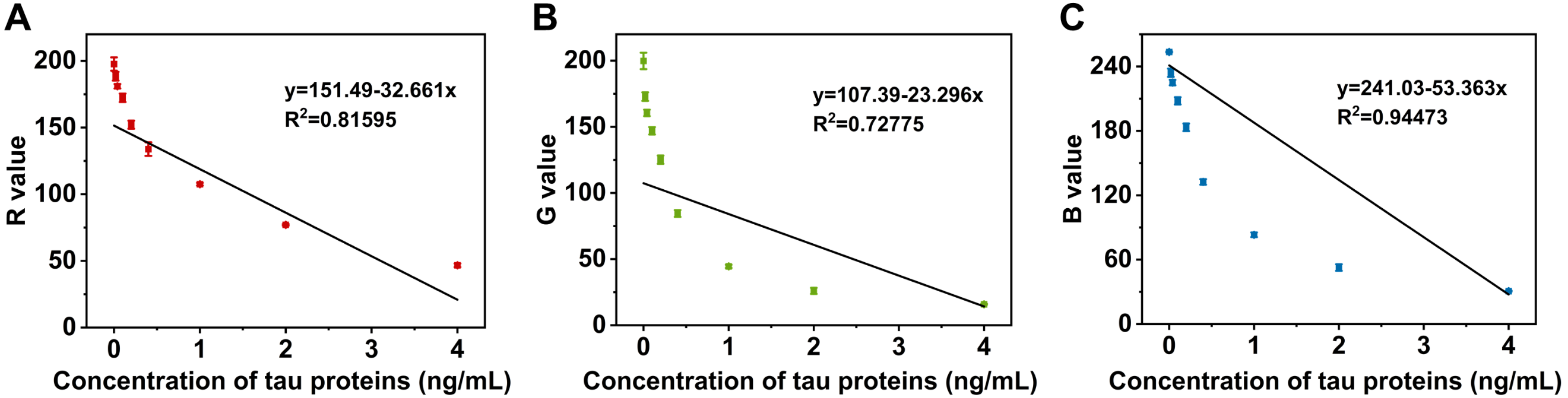


Figure S14. A) Linear fit curve of the R value to the tau protein concentration in the PBS samples. B) Linear fit curve of the G value to the tau protein concentration in the PBS samples. C) Linear fit curve of the B value to the tau protein concentration in the PBS samples.


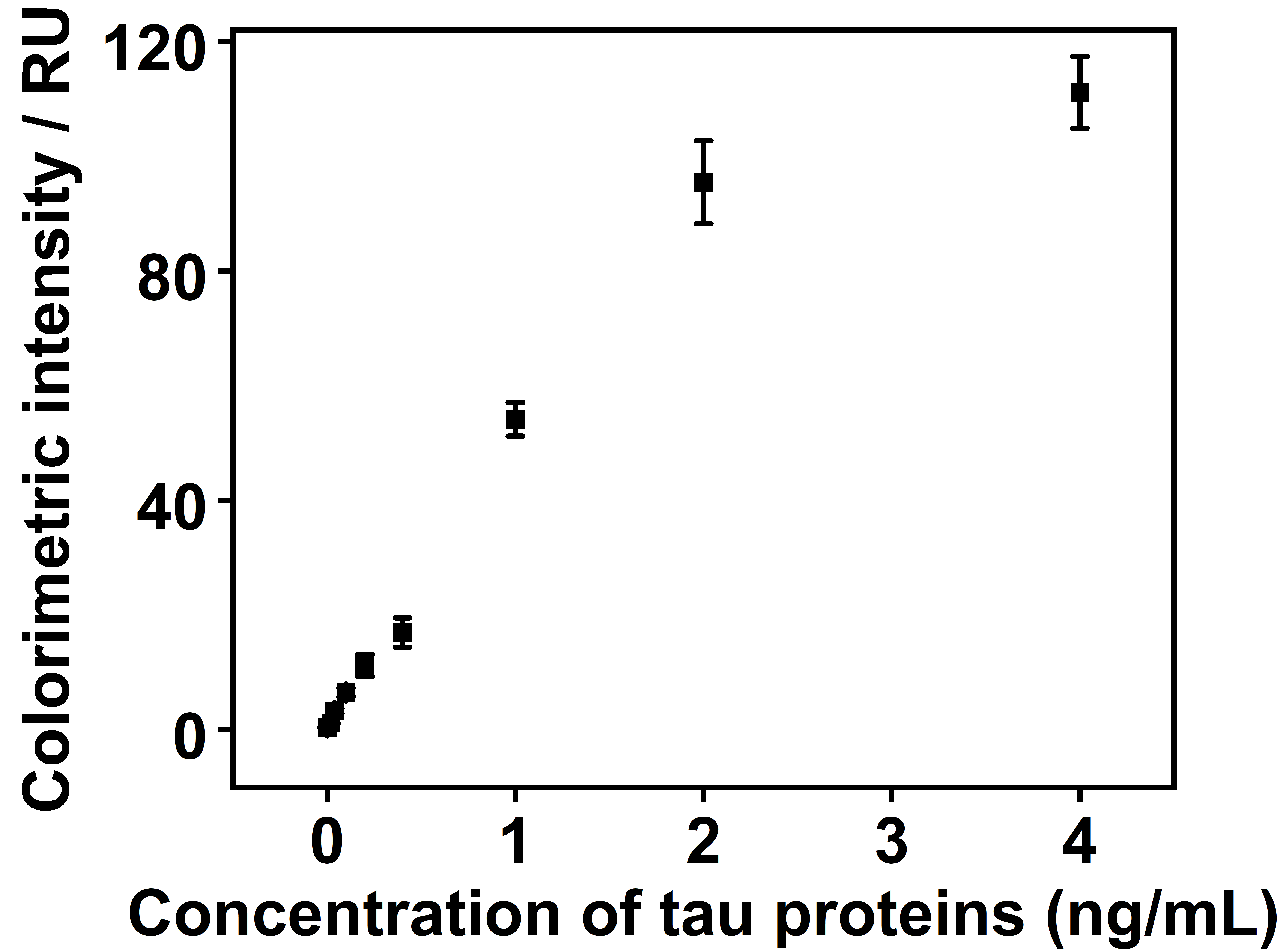


Figure S15. Scatter plot of colorimetric signals for PBS concentration gradient samples.


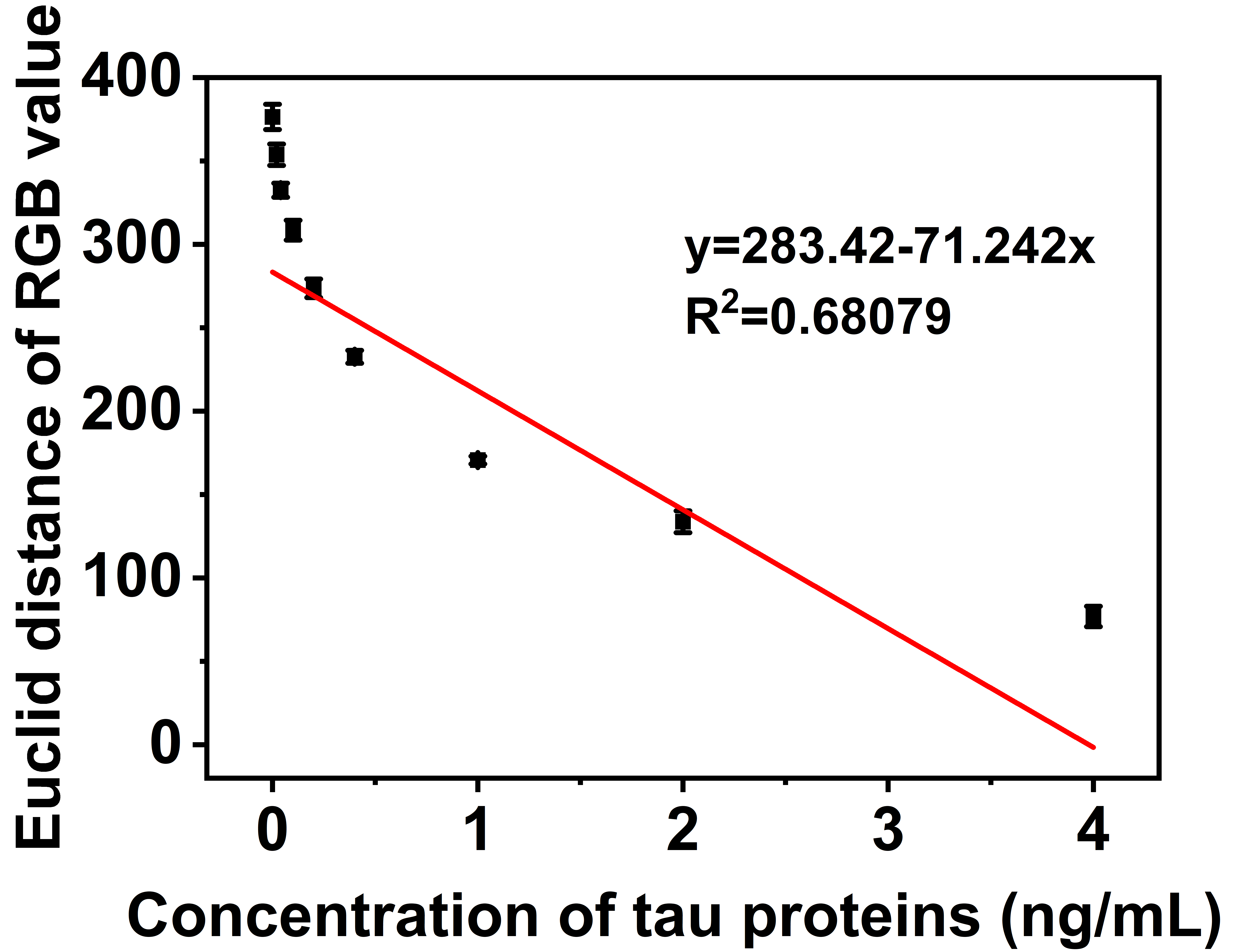


Figure S16. Linear fit curve of the RGB-valued Euclidean distance to the tau protein concentration in the undiluted serum samples.


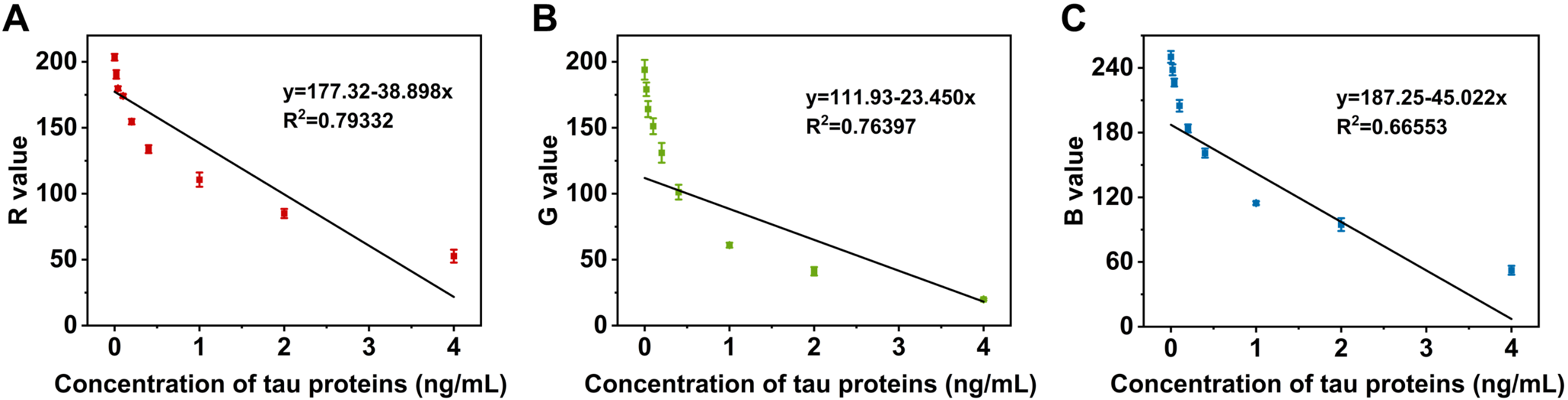


Figure S17. A) Linear fit curve of the R value to the tau protein concentration in the serum samples. B) Linear fit curve of the G value to the tau protein concentration in the serum samples. C) Linear fit curve of the B value to the tau protein concentration in the serum samples.


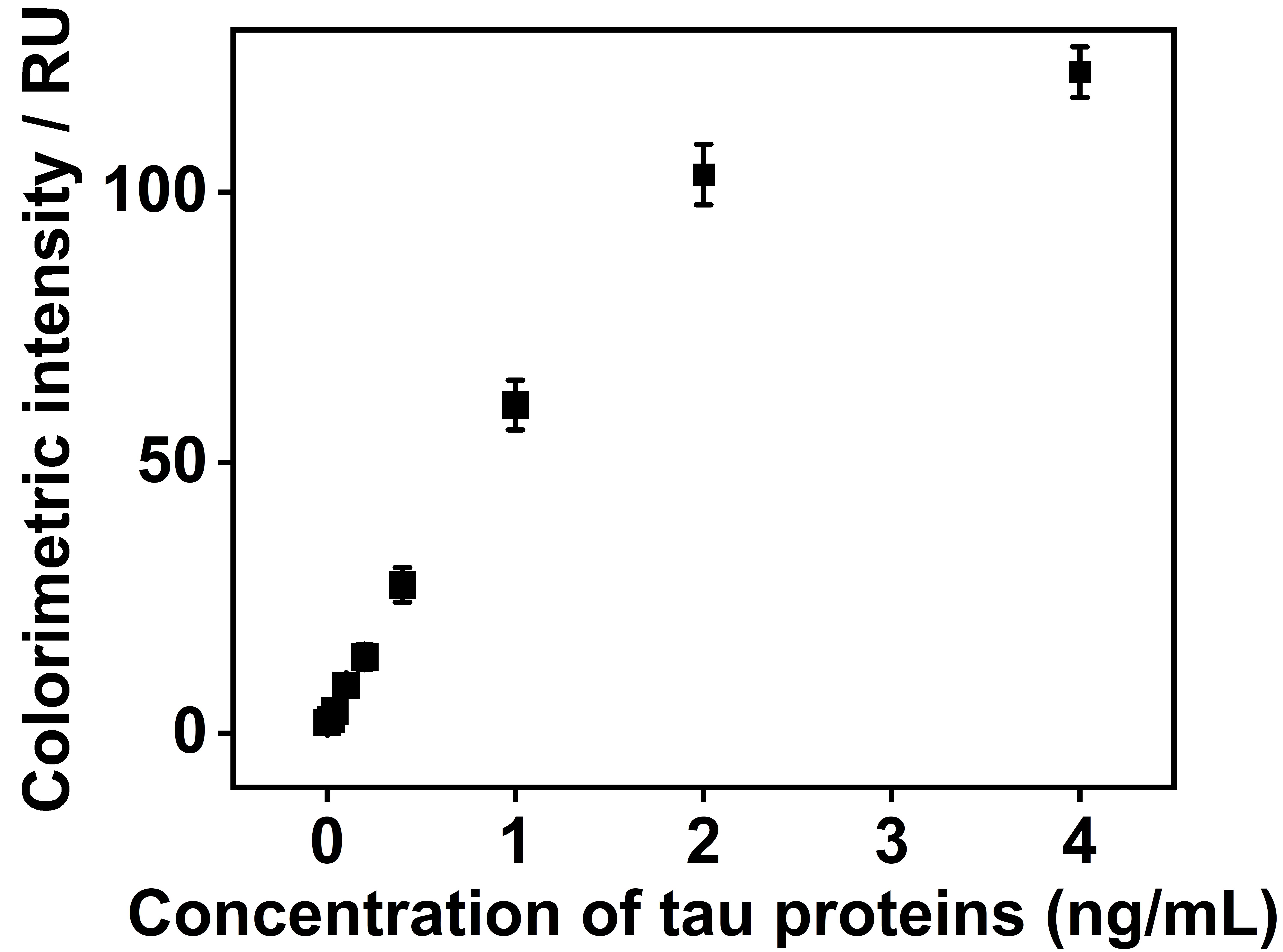


Figure S18. Scatter plot of colorimetric signals for serum concentration gradient samples.


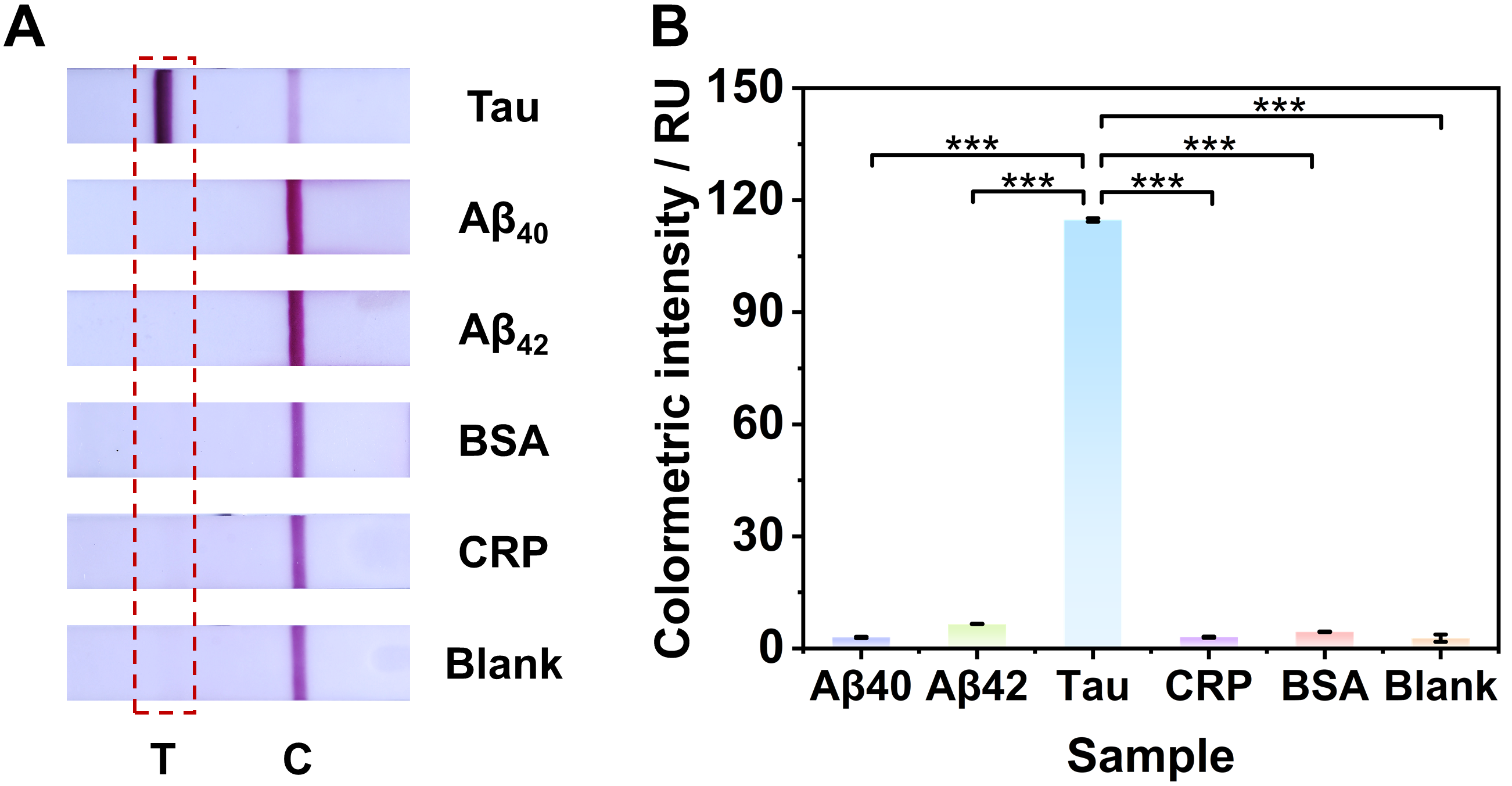


Figure S19. A) Optical images of the LFA test strips and B) specificity test of the machine learning-optimized ultrasound-enriched LFA system to target tau proteins, C-reactive protein (CRP), Aβ_40_, Aβ_42_, BSA and blank control (two-tailed Student’s t-test; ***, p<0.001). The concentrations of tau proteins, CRP, Aβ_40_, Aβ_42_ and BSA were 4 ng mL^−1^, 4 ng mL^−1^, 40 ng mL^−1^, 8 ng mL^−1^ and 4 ng mL^−1^, respectively.


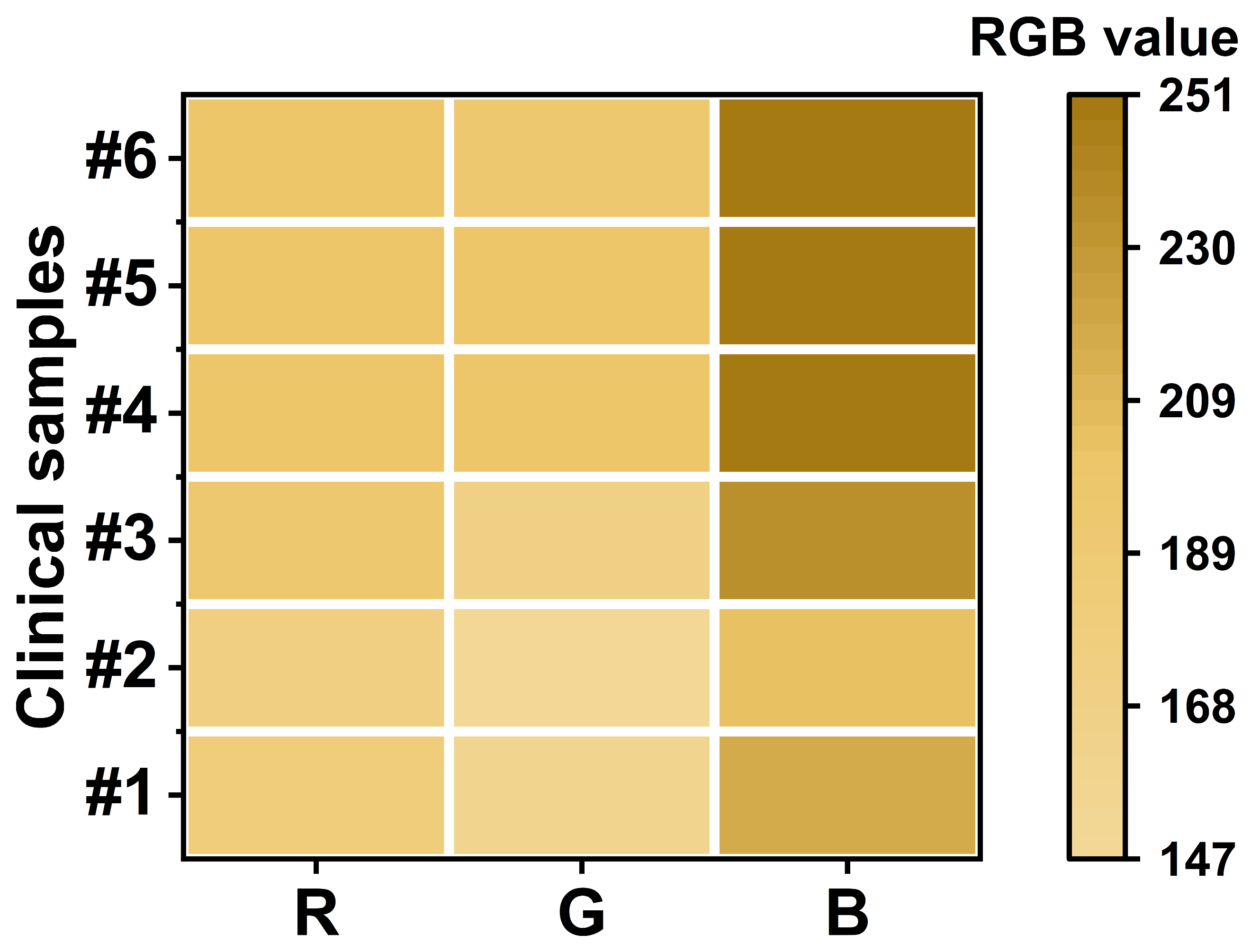


Figure S20. Heat map showing the R, G, B results of tau protein detection of the plasma samples.

Table S1. Detection of tau proteins in the PBS samples by the machine learning-optimized LFA with ultrasound enrichment (n = 3).

| **Group** | | **Spiked**  **(ng mL^−1^)** | **Colorimetric intensity**  **(RU)** | | **RSD**  **(%)** | **Calculated**  **(ng mL^−1^)** | |
| --- | --- | --- | --- | --- | --- | --- | --- |
| 1 | 0 | | 0.437±0.082 | 18.76 | | | 0.0022±0.0021 |
| 2 | 0.02 | | 1.176±0.026 | 2.21 | | | 0.0197±0.0005 |
| 3 | 0.04 | | 3.263±0.489 | 14.97 | | | 0.0601±0.0162 |
| 4 | 0.1 | | 6.522±0.771 | 11.82 | | | 0.1304±0.0248 |
| 5 | 0.2 | | 11.232±1.937 | 17.25 | | | 0.2353±0.0428 |
| 6 | 0.4 | | 16.965±2.551 | 15.04 | | | 0.3826±0.0485 |

Recovery rate (%) = C_1_/C_2_×100%

Where C_1_ represents the measured concentration in the PBS sample; C_2_ represents the spiked concentration.

Relative standard deviation (RSD, %) = SD/mean valve×100%

Table S2. Detection of tau proteins in the serum samples by the machine learning-optimized LFA with ultrasound enrichment (n = 3).

| **Group** | | **Spiked**  **(ng mL^−1^)** | **Colorimetric intensity**  **(RU)** | | **RSD**  **(%)** | **Calculated**  **(ng mL^−1^)** | |
| --- | --- | --- | --- | --- | --- | --- | --- |
| 1 | 0 | | 1.960±0.159 | 8.11 | | | 0.0025±0.0026 |
| 2 | 0.02 | | 2.517±0.272 | 10.81 | | | 0.0117±0.0045 |
| 3 | 0.04 | | 4.086±0.741 | 18.14 | | | 0.0376±0.0122 |
| 4 | 0.1 | | 8.836±1.067 | 12.08 | | | 0.1162±0.0176 |
| 5 | 0.2 | | 14.085±2.268 | 16.10 | | | 0.2029±0.0375 |
| 6 | 0.4 | | 27.397±3.216 | 11.74 | | | 0.4229±0.0532 |

Recovery rate (%) = C_1_/C_2_×100%

Where C_1_ represents the measured concentration in the serum sample; C_2_ represents the spiked concentration.

Relative standard deviation (RSD, %) = SD/mean valve×100%

Table S3. Comparison of the machine learning-optimized ultrasound-enriched LFA (ML-UELFA) with other AD detection strategies.

| **Target** | | **Method** | **POCT** | | | | **Detection time** | | | **LOD** | | **Reference** | |
| --- | --- | --- | --- | --- | --- | --- | --- | --- | --- | --- | --- | --- | --- |
| miR-101 | Electrochemistry | | | Yes | | 4 hours | | | 91.4 pM | | ^[5]^ | | |
| Aβ_42_; Aβ_40_ | Colorimetry | | | No | | 15 min | | | 2.3 nM;  0.6 nM | | ^[6]^ | | |
| miR-125b | AIE and FRET | | | No | | ≤1 hour | | | 20.81 pM | | ^[7]^ | | |
| Aβ_42_Ms; Aβ_42_Os | Colorimetry | | | Yes | | ≤30 min | | | 154 pg mL^−1^ | | ^[8]^ | | |
| Aβ_42_ | Electrochemistry | | | No | | / | | | 100 pg mL^−1^ | | ^[9]^ | | |
| Fetuin B; clusterin | Colorimetry and Electrochemistry | | | No | | ≤1 hour | | | 0.24 nM;  0.12 nM | | ^[10]^ | | |
| Aβ_42_Ms; Aβ_42_Os | Fiber-optic surface plasmon resonance | | | No | | / | | | 30 pg mL^−1^;  170 pg mL^−1^ | | ^[11]^ | | |
| tau proteins | Colorimetry | | | No | | 50 min | | | 153 pg mL^−1^ | | ^[12]^ | | |
| tau proteins | | Fluorescence | | | Yes | | 30 min | 102 pg mL^−1^ | | | ^[13]^ | | |
| **tau proteins** | | **ML-UELFA** | **Yes** | | | **10 min** | | | **10.30 pg mL^−1^** | | | | **Our work** |

The limit of detection (LOD) = S_blank_ + 3 × SD_blank_

Where S_blank_ is the average signal for a target concentration of zero; SD_blank_ is the standard deviation of the blank sample signal.

Table S4. Clinical validation of the LFA system with integrated machine learning assistance and ultrasound enrichment (AD, Alzheimer's disease patients; HC, healthy controls).

| **Sample No, type** | | **Subject information^a^** | **Chromatogram picture** | | **ML-output (ng mL^−1^)** | **Results by ML-UELFA** | |
| --- | --- | --- | --- | --- | --- | --- | --- |
| #1, plasma | 60+ years old, Female | | | 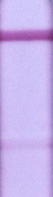 | 0.0507 | | Positive |
| #2, plasma | 60+ years old, Male | | | 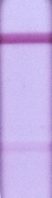 | 0.1052 | | Positive |
| #3, plasma | 60+ years old, Female | | | 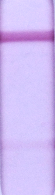 | 0.0210 | | Positive |
| #4, plasma | 60+ years old, Female | | | 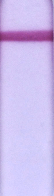 | 0.0013 | | Negative |
| #5, plasma | 60+ years old, Male | | | 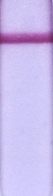 | 0.0008 | | Negative |
| #6, plasma | 60+ years old, Male | | | 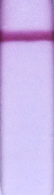 | 0.0009 | Negative | |

(a) Diagnosis by two chief physicians at Longgang District Central Hospital of Shenzhen (#1: AD; #2: AD; #3: AD; #4: HC; #5: HC; #6: HC)

**Supporting Video**

Video 1: Aggregation of AuNPs under a frequency of ~700 kHz.

**References**

[1] K. Serebrennikova, J. Samsonova, A. Osipov, *Nano-Micro Lett.* **2018**, *10*, 24.

[2] M. T. Guler, P. Beyazkilic, C. Elbuken, *Sens. Actuators, A* **2017**, *265*, 224.

[3] A. Welch-Phillips, D. Gibbons, D. P. Ahern, J. S. Butler, *Clin. Spine. Surg.* **2020**, *33*, 323.

[4] J. H. Joergensen, H. Bruus, *J. Acoust. Soc. Am.* **2021**, *149*, 3599.

[5] Y. Song, T. Xu, Q. Zhu, X. Zhang, *Biosens. Bioelectron.* **2020**, *162*, 112253.

[6] T. Hu, S. Lu, C. Chen, J. Sun, X. Yang, *Sens. Actuators, B* **2017**, *243*, 792.

[7] Q. Zhang, B. Yin, Y. Huang, Y. Gu, J. Yan, J. Chen, C. Li, Y. Zhang, S. Wong, M. Yang, *Biosens. Bioelectron.* **2023**, *230*, 115270.

[8] L. Zhang, X. Du, Y. Su, S. Niu, Y. Li, X. Liang, H. Luo, *J. Nanobiotechnol.* **2021**, *19*, 366.

[9] H. T. N. Le, J. Park, S. R. Chinnadayyala, S. Cho, *Biosens. Bioelectron.* **2019**, *144*, 111694.

[10] L. C. Brazaca, J. R. Moreto, A. Martín, F. Tehrani, J. Wang, V. Zucolotto, *ACS Nano* **2019**, *13*, 13325.

[11] L. Zu, X. Wang, P. Liu, J. Xie, X. Zhang, W. Liu, Z. Li, S. Zhang, K. Li, A. Giannetti, W. Bi, F. Chiavaioli, L. Shi, T. Guo, *Adv. Sci.* **2024**, *11*, 2308783.

[12] C. Duan, J. Jiao, J. Zheng, D. Li, L. Ning, Y. Xiang, G. Li, *Anal. Chem.* **2020**, *92*, 15162.

[13] S. Bayoumy, T. Salminen, Y. S. Hok-A-Hin, W. M. van der Flier, J. Vanbrabant, E. Stoops, E. Vanmechelen, I. M. W. Verberk, C. E. Teunissen, *Alzheimer's Dementia* **2023**, *19*, e067530.
